# Phospholipid profiling enables to discriminate tumor- and non-tumor-derived human colon epithelial cells: Phospholipidome similarities and differences in colon cancer cell lines and in patient-derived cell samples

**DOI:** 10.1371/journal.pone.0228010

**Published:** 2020-01-30

**Authors:** Jiřina Hofmanová, Josef Slavík, Petra Ovesná, Zuzana Tylichová, Ladislav Dušek, Nicol Straková, Alena Hyršlová Vaculová, Miroslav Ciganek, Zdeněk Kala, Miroslav Jíra, Igor Penka, Jitka Kyclová, Zdeněk Kolář, Alois Kozubík, Miroslav Machala, Jan Vondráček

**Affiliations:** 1 Department of Cytokinetics, Institute of Biophysics of the Czech Academy of Sciences, Brno, Czech Republic; 2 Veterinary Research Institute, Brno, Czech Republic; 3 Institute of Biostatistics and Analyses, Faculty of Medicine, Masaryk University, Brno, Czech Republic; 4 Department of Surgery, University Hospital Brno, Brno, Czech Republic; 5 Department of Anesthesiology, Resuscitation and Intensive Care, University Hospital Brno, Brno, Czech Republic; 6 Department of Pathology, University Hospital Brno, Brno, Czech Republic; 7 Department of Clinical and Molecular Pathology, Faculty of Medicine and Dentistry, Palacký University Olomouc, Olomouc, Czech Republic; 8 Department of Experimental Biology, Faculty of Science, Masaryk University, Brno, Czech Republic; University of Nebraska Medical Center, UNITED STATES

## Abstract

Identification of changes of phospholipid (PL) composition occurring during colorectal cancer (CRC) development may help us to better understand their roles in CRC cells. Here, we used LC-MS/MS-based PL profiling of cell lines derived from normal colon mucosa, or isolated at distinct stages of CRC development, in order to study alterations of PL species potentially linked with cell transformation. We found that a detailed evaluation of phosphatidylinositol (PI) and phosphatidylserine (PS) classes allowed us to cluster the studied epithelial cell lines according to their origin: i) cells originally derived from normal colon tissue (NCM460, FHC); ii) cell lines derived from colon adenoma or less advanced differentiating adenocarcinoma cells (AA/C1, HT-29); or, iii) cells obtained by *in vitro* transformation of adenoma cells and advanced colon adenocarcinoma cells (HCT-116, AA/C1/SB10, SW480, SW620). Although we tentatively identified several PS and PI species contributing to cell line clustering, full PI and PS profiles appeared to be a key to the successful cell line discrimination. In parallel, we compared PL composition of primary epithelial (EpCAM-positive) cells, isolated from tumor and adjacent non-tumor tissues of colon cancer patients, with PL profiles of cell lines derived from normal colon mucosa (NCM460) and from colon adenocarcinoma (HCT-116, SW480) cells, respectively. In general, higher total levels of all PL classes were observed in tumor cells. The overall PL profiles of the cell lines, when compared with the respective patient-derived cells, exhibited similarities. Nevertheless, there were also some notable differences in levels of individual PL species. This indicated that epithelial cell lines, derived either from normal colon tissue or from CRC cells, could be employed as models for functional lipidomic analyses of colon cells, albeit with some caution. The biological significance of the observed PL deregulation, or their potential links with specific CRC stages, deserve further investigation.

## Introduction

The colorectal cancer (CRC) development is a complex multi-step process, which involves a gradual progression from adenomatous polyp to adenoma, and then to malignant carcinoma [1]. The colon adenoma-carcinoma sequence has been suggested to be accompanied with alterations of lipid profile(s) and/or lipid metabolism, which may contribute to tumor progression, as well as to cancer cell heterogeneity [2]. This deregulation of lipid metabolism can have a major impact on both structure and function of cellular membranes, as well as on the lipid metabolism in cancer cells. Lipid alterations may thus substantially affect tumor cells behavior, including their responses to both endogenous and exogenous factors regulating their survival and/or proliferation [3, 4].

Several studies have pointed to significant changes in fatty acid (FA) composition occurring in colon mucosa during cancer development, already three decades ago [5–7]. The differences between tumor and surrounding non-tumor tissues have been later confirmed by additional observations [8–12]. CRC patients also often exhibit abnormalities of FA profiles in blood samples [13, 14]. Moreover, an inverse association of serum n-3 polyunsaturated FAs (PUFAs) and a positive association of serum n-6 PUFAs with colorectal adenoma risk has been reported [15]. Therefore, CRC development seems to be associated with changes FA profiles both in tumor tissue and in patient’s serum.

More recent studies have also indicated that increased levels of total phospholipids (PLs) and, in particular, of some specific PL classes/species could be associated with CRC development [16]. The biochemical changes involving changes in expression/activity of enzymes involved in PL synthesis, as well as an increased *de novo* lipogenesis have been shown to alter PL composition, and these might take place well in advance of morphological changes observed during tumorigenesis [17]. Interestingly, it has been also reported that distribution of some lipid species and lipid enzymes may form gradients along colon crypts, correlating either positively or negatively with the activity of Wnt/β-catenin signaling pathway and/or differentiation of colonocytes in normal colon mucosa. Importantly, this ordered distribution of lipid species/enzymes is deregulated in adenomatous polyps [18]. However, the association of specific types of PLs (or other lipids) with particular stages of CRC development, or their functional role(s) in CRC development remain largely unclear.

Recent developments in lipid analytical techniques (lipidomics) provide a unique opportunity for more sophisticated studies of the lipid alterations and their role in cancer development/therapy [2, 8, 19, 20]. However, more precise analyses of CRC lipidome will also require a better characterization of both CRC models and sample preparation. Here, colon epithelial cell lines derived from different stages of CRC development may serve as particularly useful models for investigation of molecular changes in individual lipids or lipid classes. They could be used e.g. for studies investigating possible associations of specific changes in lipid composition with modulation of proliferation, survival and differentiation of CRC cells, as well as for analyses of their responses to endogenous regulators, or to dietary and therapeutic agents [21, 22]. Some of the CRC-derived cell lines could also become valuable standards for evaluation of changes in lipidome in isolated CRC tumor cell populations. However, so far, CRC-derived cell lines have been mostly characterized with regard to their genomic alterations or specific mutations and epigenetic changes characteristic for tumor cells. We hypothesized that the results of lipidomic analyses may provide an additional level of characterization of both differences and similarities among CRC cell lines that are used in pre-clinical research. This may help to better correlate the data obtained using these cell lines, with the results acquired using isolated primary tumor cells or cells isolated from healthy colon tissue.

In the present study, we analyzed lipid composition of nine human colon epithelial cell lines derived from normal colon mucosa or from tumor tissue(s) at various stages of malignant transformation, with the aim to identify key lipid species, which would allow to discriminate among individual cell lines. We particularly focused on their PL profiles, as these have been recently shown to provide a suitable tool e.g. for discrimination between non-small cell lung cancer and normal lung tissue [23]. We then compared the obtained data with the results of evaluation of lipid composition of epithelial cells isolated from colon adenocarcinomas and adjacent non-tumor colon tissues of CRC patients. Our present results seem to suggest that permanent cell lines derived at different CRC stages can be successfully discriminated and clustered based on their PL profiles, and that, despite some limitations, they could represent useful standards for lipidomic analyses of patient-derived colon epithelial cells.

## Material and methods

### Cell culture

Human epithelial colon NCM460 cells (Incell, San Antonio, TX, USA), derived from normal colon mucosa, were cultured according to the company specifications, in M300F medium (Incell) supplemented with 10% fetal bovine serum (FBS) (Gibco, Thermo Fisher Scientific, Waltham, MA, USA) and penicillin/streptomycin (50 mg/l; Serva Electrophoresis, Heidelberg, Germany). Human fetal colon FHC cells, obtained from American Tissue Type Collection (ATCC; cat. no. ATCC® CRL-1831^™^; Rockville, MD, USA), were cultured in a 1:1 mixture of Ham's F12 and DMEM (Sigma-Aldrich; Prague, Czech Republic), supplemented with HEPES (25 mM, Sigma-Aldrich), 10% FBS, cholera toxin (10 ng/ml; Calbiochem-Novabiochem Corporation; La Jolla, CA, USA), insulin (0.005 mg/ml, Sigma-Aldrich, Prague, Czech Republic), transferrin (0.005 mg/ml, Sigma-Aldrich) and hydrocortisone (1 mg/ml; Sigma-Aldrich). Human adenocarcinoma cell lines, HT-29 (ATCC® HTB-38^™^), HCT-116 (ATCC® CCL-247^™^), SW480 (ATCC® CCL-228^™^) and corresponding lymph node metastatic SW620 (ATCC® CCL-227^™^) cell line, were cultured in McCoy's 5A medium (Sigma-Aldrich), supplemented with 10% FBS and penicillin/streptomycin (50 mg/l). Human colon adenoma AA/C1 [24], AA/C1/SB10 [24] and RG/C2 [25] cell lines were kindly provided by prof. C. Paraskeva (School of Cellular and Molecular Medicine, University of Bristol, UK). AA/C1/SB10 cell line has been established by viral *in vitro* transformation of AA/C1 cells [24]. All adenoma-derived cell lines were cultured in DMEM supplemented with 10% FBS, penicillin/streptomycin (50 mg/l), insulin (0.008 mg/ml) and hydrocortisone (1 mg/ml). Cells were routinely passaged twice a week and maintained at standard cultivation conditions (37°C; 5% CO_2_; 95% humidity). All cell lines were routinely checked for *Mycoplasma* infection.

### Preparation of samples for lipid analyses from cell lines

Cells were seeded (2×10^4^ cells per cm^2^) in cell culture dishes (TPP, Trasadigen, Switzerland). Upon reaching sub-confluency, cells were harvested and counted using Coulter Counter model ZM (Beckman Coulter, Fullerton, CA, USA). For each cell line, 8×10^6^ cells were frozen in 1 ml methanol (at -20°C) in glass tubes for lipid analyses. The tubes were previously degreased by sulfuric acid and washed with ethanol.

### Tumor tissue specimens

Fresh tumor tissues were harvested during the elective scheduled tumor removal surgery; healthy autologous tissue removed alongside the tumor was used as a non-tumor control. All tumors were generally characterized as primary moderately differentiated adenocarcinomas. All human samples were obtained at the Department of Surgery of the University Hospital Brno (Brno, Czech Republic), based on informed consent signed by patients. The study was approved by the Ethical Committee of the University Hospital Brno and all experimental procedures were in compliance with the Declaration of Helsinki, as well as with the law of the Czech Republic.

### Isolation of epithelial cells from tumors and normal colon mucosa

The surgically removed tissue was cut to smaller pieces with scissors under sterile conditions, and then incubated for 15 min at room temperature (RT) in dithiothreitol: Hank´s balanced salt solution (HBSS) (Biosera, Nuaille, France) mix (1:9 v/v), in order to remove potential contamination. Samples were then washed with HBSS (37°C), centrifuged (200g; 5 min) and digested in culture medium and TM liberase (#05401119001, Roche, Prague, Czech Republic) at 37°C for 2 h. The culture medium RPMI 1640 (#51800–035, Gibco) contained HEPES, antibiotic/antimycotic mix (#15240–062, Gibco), NaHCO_3,_ insulin, hydrocortisone and 5% FBS. After incubation, the rest of digested tissue was filtered through 50 μm filter, washed with culture medium and 20% FBS, centrifuged (200g; 5min), and finally washed with HBSS (37°C). Contaminating erythrocytes were lysed for 5 min on ice in a lysis solution (155 mM NH_4_Cl, 10 mM KHCO_3,_ 0.1 mM EDTA, ultra-pure water), and then washed with HBSS. Cell pellet was then re-suspended in a mixture of 300 μl of cold MACS buffer (PBS, 0.5% FBS, 2 mM EDTA), 100 μl FcR reagent (#130-059-901, Miltenyi Biotec, Trenčín, Slovakia), for blocking of non-specific binding of the antibody, and 100 μl magnetic beads conjugated with the EpCAM (epithelial cell adhesion molecule) antibody (#130-061-101, Miltenyi Biotec). This mixture was incubated for 30 min at 4°C in the dark, and then washed again with MACS buffer. The re-suspended cell pellet was then loaded onto magnetic column. Epithelial cells bound to magnetic beads were captured in the magnetic column and then displaced with a piston. Isolated epithelial cells were counted using Coulter Counter model ZM (Beckman Coulter, Fullerton, CA, USA) and stored in 1 ml of methanol at -20°C until further analyses.

### Lipid extraction and analyses

Cell pellets were thawed and transferred from Eppendorf tube into degreased glass tubes (100 x 10 mm) using 3 x 0.5 ml of methanol. After each addition of methanol, cell pellet was homogenized by sonication in a water bath. Following this procedure, 0.75 ml of chloroform and 0.3 ml of water were added into tube; its content was dispersed using a probe sonicator and extraction process continued at RT overnight. The monophase extraction system was dried by nitrogen at room temperature. For reconstitution of lipids, 0.3 ml of solvent mixture (methanol/chloroform 1/1 vol. %) was used. This concentrated extract was then used for liquid chromatography-tandem mass spectrometry (LC-MS/MS) analysis of lipids, performed using Agilent 1200 high performance liquid chromatography (HPLC) System (Agilent, Santa Clara, CA, USA) and mass spectrometer TripleQuad 6410, equipped with electrospray ionization (ESI) and atmospheric pressure photoionization (APPI), respectively (Agilent). Methanol, chloroform, acetonitril, hexane, isopropanol, formic acid, ammonium formate were purchased from Sigma-Aldrich; HPLC columns Supelcosil LC SI–(250 mm x 2.1 mm; 3 μm), Supelcosil LC DIOL (250 mm x 3 mm; 5 μm) were used for lipid separation.

### Liquid chromatography separation conditions

Phospholipid classes were separated by normal-phase HPLC using methanol-acetonitril-water gradient mobile phases and detected by tandem mass spectrometry with ESI. Non-polar lipid classes were separated by normal-phase HPLC using hexane-isopropanol gradient mobile phases and detected by mass spectrometry with APPI.

### Tandem mass spectrometry conditions

*Phospholipids*: ESI ionization was set in positive mode, drying nitrogen flow at 10 l/min, and fragmentor voltage and collision energy voltage were optimized for each lipid class. Neutral loss scan mode and precursor ion scan mode, respectively, were used for specific detection of respective lipid classes: precursor scan at m/z 184 for phosphatidylcholines (PC); neutral loss scan at m/z 115 for phosphatidic acids, at m/z 141 for phosphatidylethanolamines (PE), at m/z 185 for phosphatidylserines (PS), and at m/z 277 for phosphatidylinositols (PI).

*Triacylglycerols (TAGs) and cholesteryl-esters (CholE)*: APPI ionization was set in positive mode, drying nitrogen flow at 11 l/min, and fragmentor voltage was optimized for each lipid class. Detection by full scan mode were used for detection of fully separated lipid classes.

### Data acquisition and evaluation

MassHunter software (Agilent) was used for evaluation of mass spectra of all lipid classes. Peak areas of individual *m/z* species were then assigned and calculated. Levels of individual lipid species were based on their corresponding chromatographic peak areas. Results were normalized to cell numbers (per one million of cells). For this comparative method, the volume of cell extract and analyzed aliquot (i.e. injection volume) were maintained the same for all samples.

### Statistical analyses

Each PL species (represented by peak area) has specific molecular weight (MW), number of carbons (C) and number of double bonds (DB). We first verified acceptable experimental variability as the repeatability of the estimates of the content of lipid compounds in analyzed cell lines. All analyses were based on original peak area values (direct outcome from chromatograms) or on transformed peak area values (log-scale), reflecting the content of individual lipid compounds (represented by specific molecular weight species). All compared individual lipid compounds (S1 Fig) as well as their profiles represented by specific molecular weight (MW) species (S1 Fig) exhibited very low variability among estimates measured in three independent experiments. Analysis of variance (performed on log-scaled peak areas, see S1 Table) confirmed effective normalization of the peak data based on logarithmic transformation.

The results are shown for all PL classes separately, since peak areas here represent unstandardized amount across different PL classes; nevertheless, the values were fully comparable within a given PL class. In order to better display and compare amount of all PL species, peak areas in particular classes were transformed to percentiles and heat maps were built. All percentiles of amount of PL including median (i.e. 50^th^ percentile) were in the same color in all PL classes. The most discriminating species were selected using ANOVA and Tukey post-hoc test.

In case of epithelial cells isolated from patient’ s tumor (T) and non-tumor (N) tissues, the heat maps show tumor/non-tumor (T/N) cell ratio (expressed as LOG) for different PL species. Clustering using Wards method on matrix of Euclidean distances was adopted for the description of similarity of lines according to peak areas.

Principal component analysis (PCA) followed by a varimax rotation was applied for the dimension reduction and the visual description of relationship among cell lines. Position of the cell lines in the space of lipid species was computed based on their log peak values in given cell lines and lipid class. The principal components are linear combinations of the original variables (log peak areas) weighted by their contribution to explaining the variance in a particular orthogonal dimension. Principal components with eigenvalue > 1 were selected and visualized for each lipid class. PCA was performed using IBM SPSS Statistics 23 (IBM Corporation) and Statistical Software R (version 3.4.2).

## Results

### Phospholipidomic analyses of established colon cell lines

We first evaluated the content and profiles of individual PL classes, TAGs and CholE in nine human colon epithelial cell lines derived both from normal colon mucosa and from colon tumors at distinct stages of colon cancer development. These included: NCM460 cells, derived from normal adult colon mucosa; FHC cells derived from normal fetal human colon; cell lines derived from colon adenoma, RG/C2 and AA/C1 cells, as well as *in vitro* transformed variant of AA/C1 cell line, AA/C1/SB10 cells; and cell lines derived from colon adenocarcinoma—HT-29, HCT-116, SW480, as well as SW620 cell line, which has been derived from a lymph node metastasis of the same patient as SW480 cell line. A detailed description of all cell lines used in the present study is provided in Table 1.

**Table 1:** The origin of human colon epithelial cell lines used in the present study and their mutation status of cancer critical genes.

Cell line	NCM460	FHC	AA/C1	RG/C2	AA/C1/SB10	HT-29	HCT-116	SW480	SW620
**Type**	normal colon mucosa	fetal colon mucosa	adenoma	adenoma	adenoma	adenocarcinoma	adenocarcinoma	adenocarcinoma	metastasis
**Tumorigenic**	no	yes	no	no	yes	yes	yes	yes	yes
**Source; Note**	adult, M;	human fetus; 13 weeks gestation stage	adult, M; clonogenic variant of PC/AA cell line from descending colon of FAP patient	adult, F; sporadic tubular adenoma of the sigmoid colon	adult, F; in vitro-transformed variant of AA/C1 cell line	adult, F;	adult, M;	adult, M;	adult, M; lymph node metastasis
**Ploidy**	express certain genetic abnormalities (https://www.incell.com/product/ncm460d-cell-line/)	hypo-triploid [26]	aneuploid [24]	polyploid [27]	aneuploid [24]	hyper-triploid [28]	near-diploid [28]	hyper-diploid [28]	hyper-diploid [28]
**p53**	wt [29]	mutated [26]	wt [30]		wt [30]	mutated [31]	wt [31]	mutated [31]	mutated [31]
**APC**	**?**	**?**	mutated (truncated) [30]	wt [32]	**?**	mutated (truncated) [33]	wt [33]	mutated [33]	mutated [34]
**KRAS**	**?**	**?**	mutated [30]	wt [35]	**?**	wt [31]	mutated [31]	mutated [31]	mutated [31]
**BRAF**	wt [36]	**?**	**?**	wt [35]	**?**	mutated [31]	wt [31]	wt [31]	wt [31]
**PIK3CA**	**?**	**?**	**?**	wt [35]	**?**	mutated [31]	mutated [31]	wt [31]	wt [31]
**PTEN**	**?**	**-**	**?**	wt [35]	**?**	wt [31]	wt [31]	wt [31]	wt [31]

Abbreviations: APC, adenomatous polyposis coli; F, female; M, male; wt, wild type; PIK3CA, phosphatidylinositol-4,5-bisphosphate 3-kinase catalytic subunit alpha; PTEN, phosphatase and tensin homolog; ?, not known

All lipid analyses were based on peak area values (directly derived from chromatograms) reflecting the content of individual lipid compounds (represented by specific molecular weight—MW species). A wide range of MW species was detected within each PL class in each cell line. Here, our analyses aimed to identify those MW species with specific discrimination properties in particular classes. The cumulative peak areas (primary data) according to lipid species MWs and number of DB within individual PL classes, as well as neutral lipids, are shown in S2 and S3 Figs, respectively. MW was the main covariate of the total PL content in all the cell lines; the biggest differences in PC, PE and PS amount among the cell lines were generated by the content of species with MW in the range 680–830. The species with MWs higher than 830 did not seem to further contribute to overall differences. In contrast to other PLs, the most discriminating PI species were those with MW > 830 (S2 Fig). Regarding the neutral lipids, the most discriminating TAGs were species with MW 800–850; for CholE this occurred at lower MWs, between MW 600 and 650 (S3 Fig). Increment in number of DB did not significantly influence changes in the total amount of PC, PE and PS classes–their content in all compared cell lines mostly increased due to contribution of PL compounds with low degree of saturation, i.e. with 1 and 2 DB, which was similar also for TAGs and CholE. In case of PIs, PI species with higher numbers of DB (2–5) were found to contribute significantly to the total PI content.

**Fig 1:**
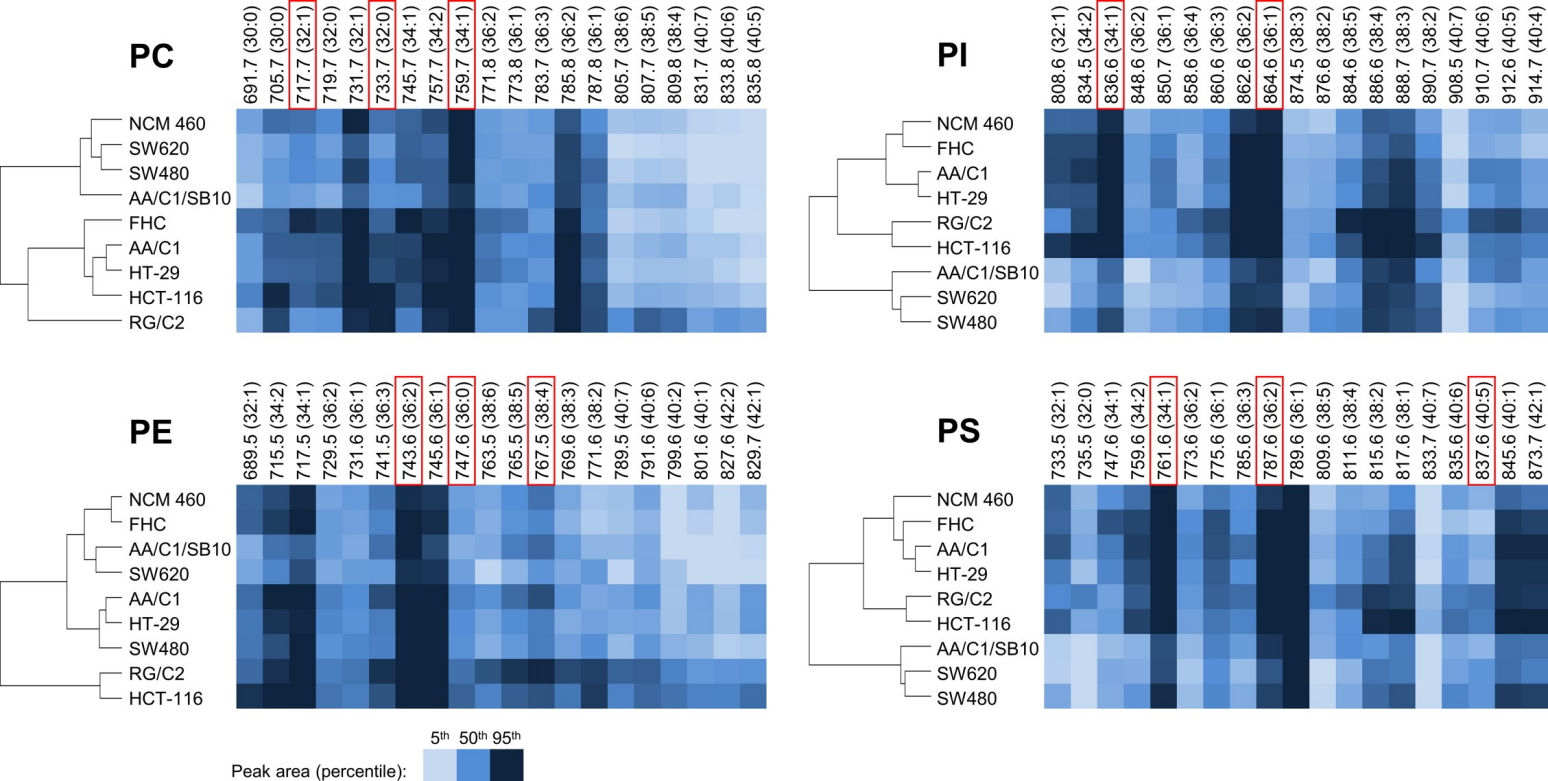
Similarity of cell lines based on the amount of specific PL species. Peak areas summarized according to lipid species MWs (shown above) as percentiles in each PL class. Carbon and DB numbers for respective MW species are shown in parentheses. Cell lines were grouped based on the results of cluster analysis (left). The color intensity reflects the abundance of the respective PL species in cell line (see the color key in the Figure). PLs with the largest differences among cell lines (the most discriminating) are highlighted in red frames.

**Fig 2:**
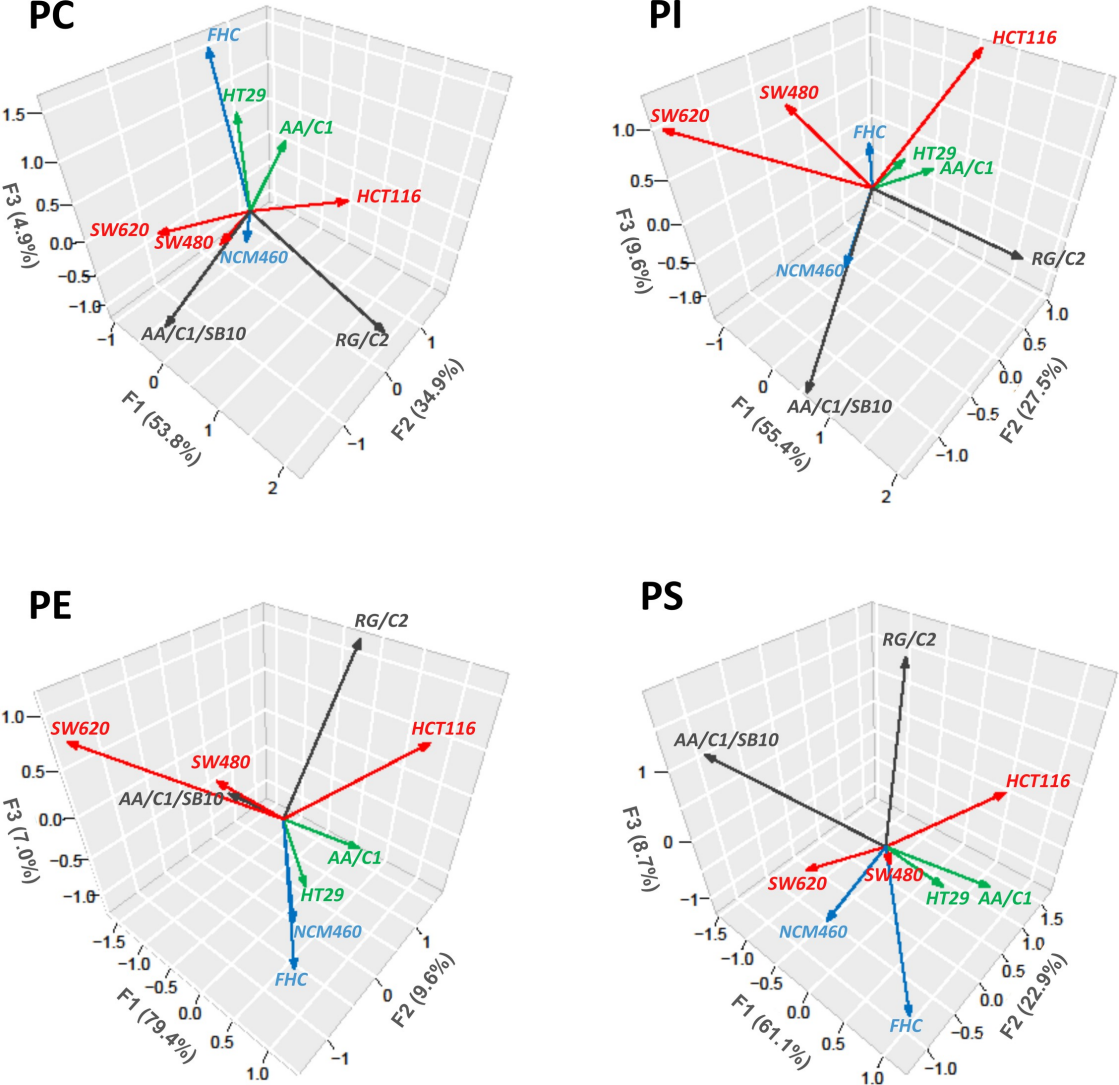
Principal component analysis (PCA) of PL species amount in colon cell lines. Phosphatidylcholine (PC): The first component (F1) is strongly correlated with species 36:3 (MW 783.7) and other 6 species with MW > 800 (see Fig 3), i.e. the first principal component increases with increasing species with 3 or more DBs. This suggests that these species exhibit a common trend–if one increases, then the remaining ones tend to increase as well. The component F2 is correlated with species with MW <733. The component F3 correlates with species 34:1, 36:2 and 36:1. These three components explain 93.6% variation of the PC data in total. Phosphatidyletanolamine (PE): The first component is strongly correlated with species with MW <720, 747.6 and 829.7 and explains almost 80% variation of the data. F2 correlates with species with ≥3 DBs and F3 with species 771.6 (38:2) and 799.6 (40:2). Phosphatidylinositol (PI): Three components explain >90% variation of the data; all examined PI species are strongly correlated with one of these 3 components. Species with 3 or more DBs correlates with F1; species with MW<865 and with 1 or 2 DBs are included in F2, whereas F3 is strongly correlated with remaining 38:2 species (MW 876.6 and 890.7). Phosphatidylserine (PS): F1 correlates with low MW species (<780), F2 correlates with higher MW species (≥815) and F3 with middle MW species, especially PS 38:4 and 38:5. For more details and potential FA composition of individual MW species, see S2 Table.

We then focused on a more detailed evaluation of individual PL classes profiles (individual MW species). A summary of suggested FA patterns in all evaluated PL species is presented in S2 Table, indicating number of carbons (C) and DB. The data were further subjected to cluster analysis (Fig 1) as well as PCA **(**Fig 2**)**. Contributions of specific lipid MW species are graphically depicted in form of heat maps (Fig 1**)**, where color intensity matches a relative contribution of a given species to the particular PL class content. Here, we were able to identify PL species with the highest discrimination power (i.e. with the largest differences observed among the cell lines), that are marked in red in Fig 1. Importantly, the cluster analysis of distribution of PI and PS species in individual cell lines grouped together cell lines derived from normal colon mucosa (NCM460, FHC) or from adenoma or a less advanced tumor (AA/C1 and HT-29) vs. cells derived from adenocarcinomas or transformed AA/C1 variant (SW480, SW620, AA/C1/SB10). Adenocarcinoma HCT-116 cells (which segregated together with adenoma RG/C2 cells) seemed to be closer to the first group. In contrast, the clustering of cell lines based on PE and PC analyses did not reveal such a clear relationship among cell lines.

PCA in Fig 2 describes the data in a fewer variables (principal components different for particular PL class and specified in the legend), which correspond to original values (log peak areas of all species). In PCA, AA/C1 and HT-29 cell lines segregated together for all PLs. NCM460 and FHC cell lines, both derived from normal tissues, had similar PE and PS profiles (content of individual species), but distinct PC and PI profiles. In addition, when we performed PCA for PE and PS species, both cell lines (NCM460, FHC) were closer to AA/C1 and HT-29 cell lines (derived from adenoma or less advanced carcinoma). This trend was less obvious in case of PC and PI species. The results of PCA confirmed that transformed variant of adenoma cells (AA/C1/SB10) is clearly distinct from parental cells (AA/C1), regarding their PL species content. SW620 and SW480 lines mostly segregated together in PCA, especially in case of PC and PI species, which may reflect their common origin. Similar to the results of cluster analysis, carcinoma HCT-116 cell line seemed closer to RG/C2 adenoma cell line, regarding to their PS or PE content. Together, the results of both cluster analysis and PCA seem to indicate that full PL (in particular PS) profiles may help to discriminate between cell lines derived either from normal colon mucosa and/or less advanced colon cancer stages on one side vs. advanced colon adenocarcinomas on the other side.

### PL profiling of primary epithelial cells isolated from colon tumors and from corresponding normal colon tissues

**Fig 3:**
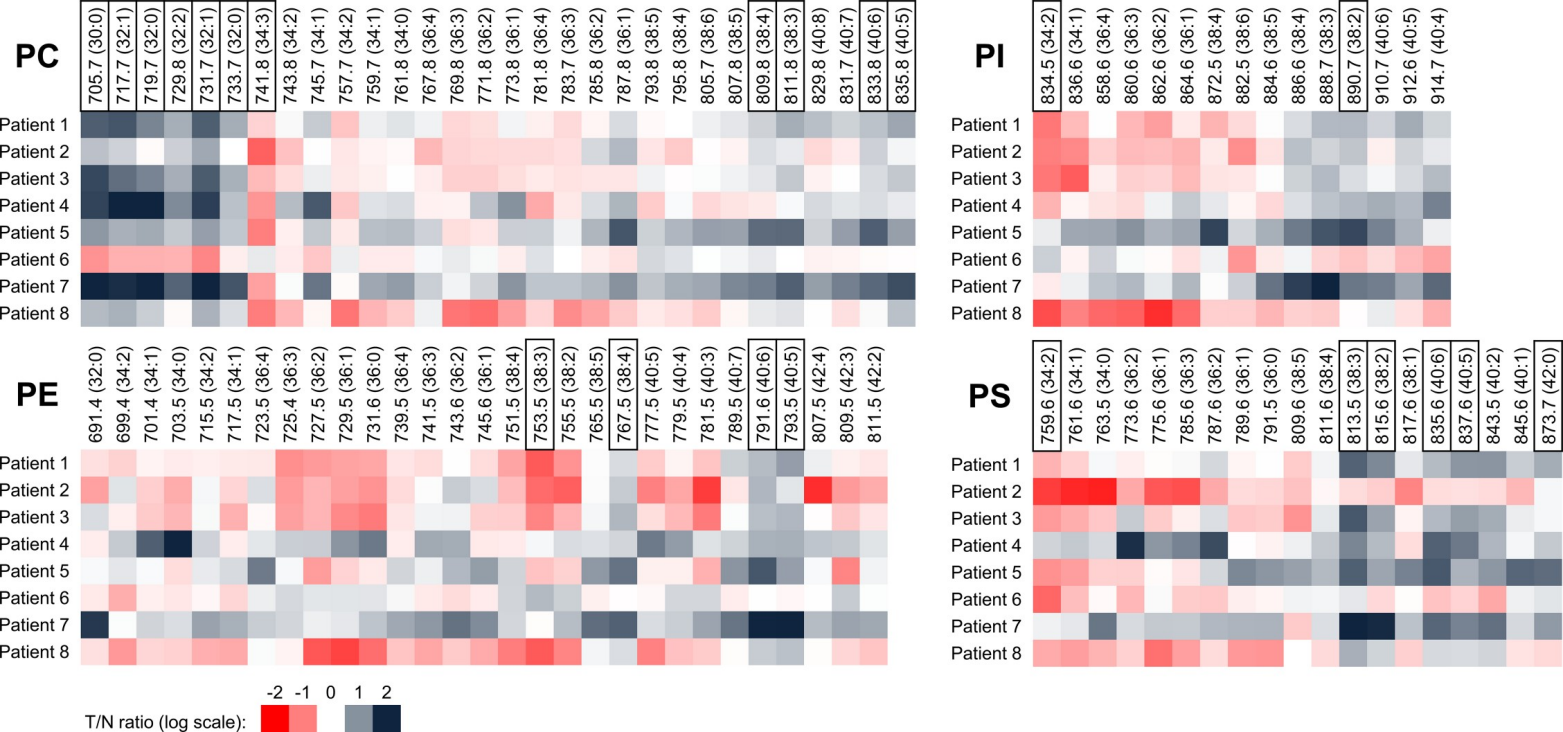
Tumor/non-tumor (T/N) ratios of specific PL species in patient-derived primary epithelial cells. T/N ratio of chromatogram peak areas summarized according to MWs of lipid species (log transformation) in CRC patients (n = 8). Carbon and DB numbers are shown in parentheses. White color (zero value)—no difference between peak areas (= lipid amount) in tumor and non-tumor cells of the patient. Values lower than zero (red color)—lower amount of the respective PL species in tumor cells as compared with non-tumor cells. Values higher than zero (grey color)—higher amount of the respective PL species in tumor cells as compared with non-tumor cells. PL species with significant difference between tumor and non-tumor cells (the most discriminating) are highlighted in black frames (paired *t*-test).

The same analytical approach, which was used for colon cell lines, was then applied for analysis of PL content of epithelial cells isolated from tumor (T) and adjacent non-tumor (N) tissues from eight CRC patients. The heat maps, based on the T/N ratio values for individual lipid MW species of PC, PI, PE or PS classes, are presented in Fig 3. In general, the PL content was higher in tumor than in non-tumor cells. However, significant differences were observed in the content of individual PL species within all PL classes, when comparing tumor and non-tumor cells. Here, several trends were discernible. Table 2 summarizes PL species with the most significant differences between tumor and non-tumor cells (average values of 8 patients), including the information on their hypothetical FA composition. In general, these PL species were mostly significantly higher in tumor cells (marked in bold); nevertheless, a few PL species were more abundant in non-tumor cells (namely, PC 741.8, PE 753.5, PI 834.5 and PS 759.5). The most discriminating PC species (eight) included both lower MW species (MW 705.8–741.8), containing short saturated and/or mono-unsaturated FA chains (C 30–34, 0–2 DB), and higher MW species (809.8–835.8) containing longer and more unsaturated FA chains (C 38–40, 3–6 DB). In PE profile, four discriminating higher MW species with longer-chain FAs (C 38–40, 4–5 DB) were identified. We found only one PI species (MW 890.5, C 38, 2 DB) to be significantly increased in tumor cells. Finally, in the PS profile, there were six significantly discriminating species–mostly with higher MWs (813.5–873.5), containing longer-chain unsaturated FAs (C 38–42, 1–6 DB).

**Table 2:** Summary of different mean peak areas between tumor (T) and non-tumor (N) cells (the most discriminating species).

MW	T/N ratio Mean (95% CI)	p-value*	FA pattern			
**PC**						
**705.7**	**2.97 (1.47–6.03)**	**0.019**	**12:0//18:0**	**14:0//16:0**		
**717.7**	**2.83 (1.5–5.34)**	**0.015**	**14:0(pla)//18:1**	**16:0(pla)/16:1**		
**719.7**	**2.49 (1.25–4.96)**	**0.036**	**14:0(pla)//18:0**	**16:0(pla)/16:0**		
**729.8**	**1.71 (1.1–2.64)**	**0.047**	**14:0//18:2**	**16:1//16:1**		
**731.7**	**2.93 (1.47–5.85)**	**0.019**	**14:0//18:1**	**16:0//16:1**		
**733.7**	**1.72 (1.19–2.47)**	**0.022**	**14:0//18:0**	**16:0//16:0**		
741.8	0.5 (0.37–0.69)	0.004	14:0(pla)//20:3	16(pla)//18:3		
**809.8**	**1.6 (1.11–2.3)**	**0.039**	**18:0//20:4**	**18:3//20:1**	**18:2//20:2**	**18:1//20:3**
**811.8**	**1.78 (1.2–2.64)**	**0.024**	**20:1//18:2**	**20:0//18:3**	**20:3//18:0**	
**833.8**	**1.76 (1.19–2.6)**	**0.026**	**20:3//20:3**	**20:4//20:2**	**20:5//20:1**	
**835.8**	**1.79 (1.24–2.59)**	**0.017**	**20:1//20:4**	**20:2//20:3**	**20:0//20:5**	
**PE**						
753.5	0.57 (0.34–0.95)	0.067	14(ple)//24:3	16(ple)//22:3	18(ple)//20:3	
**767.5**	**1.84 (1.3–2.61)**	**0.011**	**20:4//18:0**	**20:1//18:3**	**20:3//18:1**	
**791.6**	**2.28 (1.47–3.54)**	**0.008**	**20:3//20:3**			
**793.5**	**2.24 (1.32–3.8)**	**0.021**	**20:1//20:4**	**20:0//20:5**	**18:0//22:5**	
**PI**						
834.5	0.56 (0.35–0.89)	0.043	16:0//18:2	16:1//18:0		
**890.7**	**1.78 (1.12–2.83)**	**0.046**	**18:0//20:2**	**18:1//20:1**	**18:2//20:0**	**16:0//22:2**
**PS**						
759.5	0.53 (0.34–0.82)	0.026	16:0//18:2	16:1//18:1		
**813.5**	**2.89 (1.65–5.06)**	**0.008**	**20:1//18:2**	**20:0//18:3**	**20:3//18:0**	
**815.6**	**1.88 (1.12–3.16)**	**0.049**	**20:2//18:0**	**20:1//18:1**	**22:1//16:1**	**22:2//16:0**
**835.6**	**1.93 (1.13–3.28)**	**0.046**	**20:3//20:3**	**22:6//18:0**	**22:5//18:1**	
**837.6**	**1.79 (1.18–2.72)**	**0.029**	**20:1//20:4**	**22:5//18:0**	**22:4//18:1**	
**873.7**	**1.57 (1.09–2.28)**	**0.048**	**22:1//20:0**	**22:0//20:1**	**24:0//18:1**	**24:1//18:0**

MW, molecular weight; CI, confidence interval of mean; significantly up-regulated species in tumor cells are shown in bold

* paired t-test, p-values were not corrected for multiple comparison

### Comparison of PL profiles between primary epithelial cells and colon cell lines

**Fig 4:**
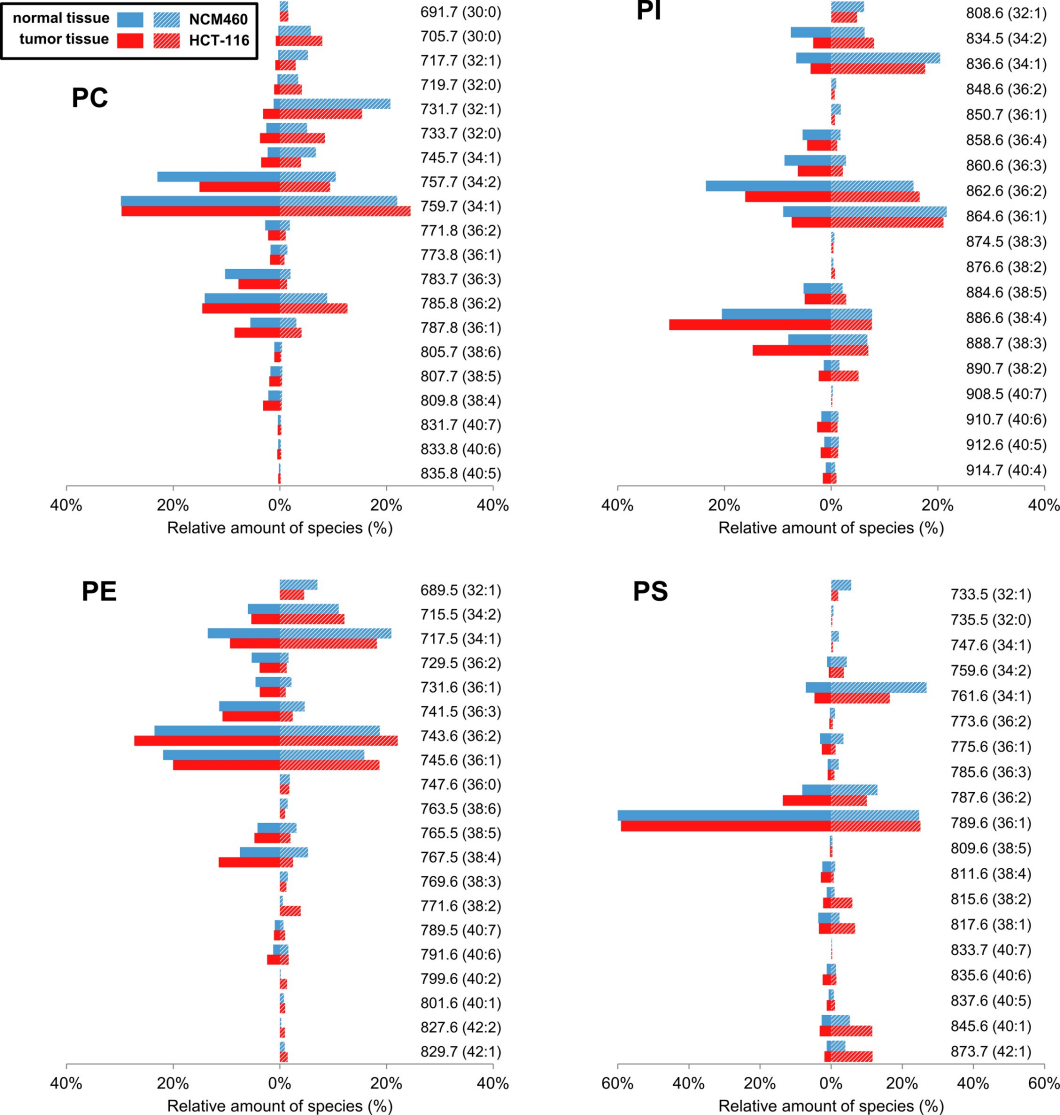
Comparison of PL profiles between patient-derived primary cells and NCM460/ HCT-116 cell lines. Relative distribution (i.e. sum of all shown MW species gives 100%) of specific PL species in non-tumor and tumor primary epithelial cells (mean value, n = 8) as well as non-tumor (NCM460) and tumor (HCT-116) derived cell lines. Carbon and DB numbers are shown in parentheses. Only PL species, which were above detection limit both in patient’s samples and in cell lines are shown here.

Based on the above results, we then compared individual PL class composition (PL profiles) that were determined in patient’s non-tumor and tumor cells, with PL profiles of model cell lines derived from normal colon mucosa (NCM460 cells) and malignant adenocarcinoma (HCT-116 cells). HCT-116 cell line was selected for this comparison, because it is a widely used colon carcinoma model, with a number of available transgenic variants, which is capable to form tumors and metastases *in vivo* with a high efficiency [37]. Relative distribution (where the sum of all shown MW species represents total content of given PL class, i.e. 100%) of specific PL species within PC, PI, PE and PS classes, which were identified both in the two analyzed cell lines and in primary cells from patients, is summarized in Fig 4. The same analysis was then also performed with another adenocarcinoma model, SW480 cells, and its results were largely similar (S4 Fig). In general, distribution of these PL species in colon cell lines seemed to correspond well with their profiles in primary epithelial cells. The most abundant PL species in patient-derived samples were also the most abundant ones in the colon-derived cell lines. Nevertheless, a similar overrepresentation of PL species in tumor vs. non-tumor cells, as in adenocarcinoma vs. normal mucosa-derived cell line, could only be observed for some PL species. These included: PC (MW 705.7 and 733.7), PI (MW 890.7) and most notably, several PS species (MW 815.6, 837.6, 845.6 and 873.7). In addition, a common down-regulation was observed for PS species with MW 759.6 and 761.6, and this was found also for some other less discriminating species not listed in Table 2. Nevertheless, it should be also noted that for some species, opposite trends could be also observed, e.g. for PC with MW 731.7 (its levels were higher in NCM460 cells than in HCT-116 or in SW480 cells, while the same species increased in tumor cells as compared with adjacent non-tumor cells), PI MW 862.6 (decreased in tumor cells but found at similar quantities both in NCM460 cells and in CRC cell lines), or PS MW 787.6 (higher in primary tumor cells, but lower in CRC cells lines). Together, these data confirmed that overall PL profiles are similar between primary cells and cultured cell lines; however, some individual PL species could be differentially regulated in primary cells as compared with CRC-derived or immortalized colon epithelial cells.

## Discussion

An altered lipid composition can have a major impact on survival, proliferation or communication of tumor cells, due to the multiple roles of lipids in cellular signaling, build-up of cell membranes or in cell-to-cell interactions [20, 38]. CRC development has been shown to be associated with a number of lipid alterations (reviewed in [39]), including increasing amounts of some PLs in transformed cells [40–42]. For example, Li *et al*. established correlations of increased PI and PC with CRC genesis, as well as the relationship between increased PE and hepatic metastasis in colorectal carcinoma [43]. Nevertheless, a detailed evaluation of changes of individual PL species during CRC development, in particular in cell lines isolated at distinct stages of colon adenoma–adenocarcinoma sequence, is still largely missing, although these cell lines may represent valuable tools for development of an improved CRC diagnostics and therapy. Recent advances of PL profiling in other cancer types [23, 44] have led us to examine in a detail PL profiles of a battery of cell lines derived from normal colon mucosa and from various colon adenoma/adenocarcinoma cells. We then continued to analyze individual PL profiles in primary cells isolated from normal and tumor tissue of CRC patients. Currently, most of the available data on differences between PL content in tumor and non-tumor tissues is based on studies evaluating lipid content in whole tumors. However, these samples may contain also significant quantities of immune cells, endothelial cells or other cancer-associated cells, such as fibroblasts, and the results of these analyses may thus not always properly reflect PL profiles of tumor epithelial cells. Hence, in the present study, we used epithelial cells isolated directly from dissociated tumors and from the respective adjacent non-tumor colon tissues of CRC patients, based on their surface EpCAM (CD326) antigen expression [45].

The results of this study seem to suggest that the commonly used human colon epithelial cell models can be discriminated based on their PL (in particular PS and PI) profiles. A detailed analyses of individual MW species within given both PS and PI classes revealed that cell lines can be segregated together in an order corresponding with their origin, i.e. into cells originally derived from normal colon tissue (NCM460, FHC), from colon adenoma or less advanced differentiating adenocarcinoma cells (AA/C1, HT-29) on one side, and, into cells obtained by *in vitro* transformation of adenoma cells and/or advanced colon adenocarcinoma cells (HCT-116, AA/C1/SB10, SW480, SW620) on the other side. A notable exception among the studied cell lines was RG/C2 cell line that has been originally derived from adenoma cells and which co-segregated with adenocarcinoma HCT-116 cells. However, this adenoma cell line has been previously shown to exhibit a relatively high malignant potential [25], unlike other adenoma-derived cell lines, thus suggesting that its PL profile could indeed be closer to non-differentiating adenocarcinoma HCT-116 cells, than to AA/C1 or HT-29 cell models. This observation appears to be supported also by the results of PCA analysis, where both RG/C2 and AA/C1/SB10 cells were separated from other cell lines. Overall, the present data seem to imply that changes in PI and PS profiles can be expected to reflect the degree of transformation of CRC cells. Although PI and PS are less abundant than PC or PE, they could play important signaling or regulatory roles in cells. Full PI and PS profiles appeared to be a key to the cell line discrimination, while FA composition of discriminating MW species appeared to be specific for individual PL classes, as shown in Table 2. Nevertheless, the changes were not limited to PS and PI classes, as we have observed also significant alterations of other PL species.

Significant differences in the abundance of lipid species have been found to be associated not only with formation of primary tumors, but also with further cancer progression and formation of metastases. For example, increased plasmanylcholine and TAG levels and decreased plasmenyl-ethanolamine lipids have been observed in metastatic SW620 cells, as compared to the primary colon cancer SW480 cell line [46]. In the present study, we found significantly increased CholE and TAG levels in SW620 cells as compared with SW480 cell line. This is in line with the evidence suggesting that the upregulation of cholesterol synthesis in metastatic cells alters signaling functions of lipid rafts, and that it is a part of general changes in lipid metabolism, including also FA synthesis, transport, or their oxidation [47]. In contrast, we observed that both SW480 and SW620 cells were rather similar in terms of their PL profiles, as suggested by PCA. However, specific PL profiles, as well as accumulation of some PL species (in particular PS, PI and PE) have been proposed to be linked with metastatic potential and aggressiveness of tumor cells in other cancer types, including breast cancer or melanomas, or to increase directly in metastatic cells [44, 48–51]. This would suggest that, in addition to general alterations of lipid metabolism, PL can be expected to increase also in colon cancer metastases. Here, we observed only minor differences between SW480 and SW620 cells in the present study, in terms of their PL profiles; however, our analysis here was limited to only one primary tumor-metastasis pair of isogenic cell lines, only minor observed differences could be in part cell-specific. Also, production of PLs might be different under *in vivo* conditions. For example, it can be expected that *in vivo*, colon cancer metastatic cells could also increase FA import from metastatic niche and consequently also PL production, e.g. through CD36 FA transporter up-regulation [47]. PL profiling of colon cancer-derived metastatic cells thus certainly deserves more attention, as it may contribute to our understanding of these highly aggressive tumor cells.

Regarding the mechanisms responsible for the observed alterations of PL profiles, several hypotheses have been proposed. Recently, the differences in lipid profiles and key lipid metabolism enzymes have been compared between DNA mismatch repair-proficient and -deficient colon cell lines. In mismatch repair-proficient cell lines (SW480, SW620, HT-29, NCM460), higher levels of PC (16:0/18:1) and phosphatidic acid (18:0/18:0) were detected, as compared with mismatch repair-deficient cell lines (HCT-116, DLD1, LoVo, HCT15) [52]. Nevertheless, it cannot be excluded that distinct PL profiles could be, at least in part, linked to the ability of colon epithelial cells to differentiate. Specific PL species seem to be tightly regulated during differentiation of colon cells, which plays a key role in maintenance of the colon crypt homeostasis. Recently, it has been reported that the turnover of arachidonic acid-containing PL species (mainly PI), is a highly synchronized event during colonocyte differentiation, and that the gradient of lipids and enzymes involved in lipid mobilization, which is observed in healthy colon crypts, seems to be largely lost in adenomatous polyps, representing the first step in CRC development [18]. Changes in PL profiles were also observed during cell differentiation of breast or kidney cells [38, 53]. Therefore, distinct PL profiles observed between non-differentiating adenocarcinoma-derived cell lines and other colon-derived cell models may also reflect disruption of proliferation/differentiation balance, and this might have consequences both for transformation of colon epithelial cells and their responses to dietary factors or treatment [54].

Our subsequent analyses of isolated primary colon epithelial cells isolated from CRC patients, detected higher levels of PLs of all classes in tumor than in non-tumor samples. This was most evident for PC, the principal PL component of cell membranes. Our results are mostly in a good accordance with earlier studies using complete colon tumor tissues [11, 40, 41]. The increased PL content may be associated with enhanced membrane synthesis accompanying accelerated cancer cell proliferation. Indeed, PC, as well as choline metabolites derived from its synthesis and catabolism, contribute to regulation of cell proliferation and survival [55]. Increased levels of specific PLs have been observed also in other cancer types, including lung, ovarian, breast, prostate, bladder, kidney, or thyroid cancer (reviewed in [16, 20]). However, the major finding of our study is the importance of full MW profiles of individual PL classes for discrimination between tumor and non-tumor cells. We then also identified as discriminating a specific subset of lipid species within a particular PL class. Within PC, saturated and monounsaturated lower MW species, as well as higher unsaturated MW species were significantly increased in tumor epithelial cells (for their expected FA composition, see S2 Table). In case of PE and PS, some species with higher MWs were significantly increased in tumor cells, although the degree of their unsaturation varied. For PI, inverse significant differences in two MW species corresponding to composition 18:0/20:2 (higher in tumor cells) and 16:0/18:2 (higher in normal cells) were identified. Interestingly, also in other PL classes we detected some PL species that were reduced in tumor cells (see Table 2). These results obtained with isolated EpCAM-positive cells can be presently compared only with the studies using whole tumor tissues. Increased levels of PC (16:0/16:1) have been found in tumor tissue of CRC [42]. Here, we have also observed increased PC with MW 731.7 (which corresponds to PC 16:0/16:1). Using a similar analytical technique, MALDI imaging mass spectrometry, Mirnezami et al. have reported elevated levels of PC 16:0/18:1 in colon tumor samples [56]; however, this particular PC was not among the most altered PL species in our samples.

Some discriminating species in our study also contained similar saturated and monounsaturated acyl chains. Increased levels of such PLs could be attributed to increased endogenous FA synthesis due to overexpression of key enzymes (FA synthase and stearoyl-CoA desaturase) in cancer tissue, including colon cancer microenvironment [57]. Nevertheless, we detected also significantly discriminating PC, PE and PS species containing longer and more unsaturated acyl chains. This is of interest, as several PE species, such as PE (38:6) or PE (40:4), have been reported to be up-regulated in CRC tissues as compared to normal tissues [58].

Alterations of PL profiles in cancer cells have been shown to promote activity of pro-survival signaling pathways or to modulate their invasivity [59, 60]. This is particularly relevant for PIs, which play some key roles in membrane dynamics and signal transduction pathways. Here, enrichment with specific acyl chains may contribute to their specificity, as e.g AA-containing PIs oscillate during the cell cycle and inhibit Akt kinase membrane binding, thus suppressing cell proliferation [61]. PS plays a key intracellular function as the precursor of PE; however, it also modifies catalytic activities of several key signaling proteins such as dynamin-1, protein kinase C or annexin V, and PS exposure on cell surface represents a signal for the recognition of apoptotic cells by phagocytic cells [62].

Finally, we found that the most abundant PL species in primary epithelial cells were also the most abundant PL species found in model colon cell lines. Thus, when we compared individual PL profiles of the two studied cell lines (NCM 460 with HCT-116 or SW480 cells) with patient’s derived primary non-tumor and tumor epithelial cells, we observed, in spite of overall higher PL levels in cell lines, significant similarities between overall PL profiles of a cell lines and the respective clinical material. Thus, epithelial cell lines derived either from normal colon tissue or from CRC cells could be employed as models for functional lipidomic studies in colon cells. Nevertheless, it should be stressed that, although we identified several specific PL species to exhibit similar ratios in tumor vs. non-tumor cells, as in NCM460 vs. adenocarcinoma cell lines, there were also some PL species exhibiting opposite trends in primary colon cells and in permanent colon cell lines, including PC MW 731.7, PI MW 862.6 or PS MW 787.6. This suggests that colon cell lines as models for lipidomics may also have some limitations (as discussed above in case of metastatic SW620 cell line). For example, changes associated with immortalization of non-tumorigenic colon cells (such as NCM460 cells) could also partly alter their PL profiles. Therefore, the data from *in vitro* cell lines should be interpreted with caution and, ideally, these experiments should be combined with analyses of primary colon cells for validation of the cell line-derived data.

In conclusion, PL profiling was found to allow for a successful discrimination among colon epithelial cell lines derived from normal colon tissue, or, at various stages of cell transformation. The present results confirm that cell transformation may lead to a significant deregulation of some PL species, which is reflected also in their differences among various colon cell lines. Importantly, for the first time, we performed a detailed analysis of PL profiles in isolated primary epithelial (EpCAM-positive) colon tumor cells from CRC patients and compared them with adjacent non-tumor epithelial cells isolated from the same donor. Here, we found increased levels of all PL classes in primary tumor cells, and we identified some specific lipid species within their profiles, with a potential to distinguish between tumor and non-tumor cells. The overall similarities of PL profiles between colon cell lines and isolated colon epithelial cells also suggest that permanent colon cell lines could serve as suitable models for lipidomic analyses. Nevertheless, when investigating the PL-dependent mechanisms regulating colon cancer cell survival, proliferation and differentiation, or, when evaluating therapeutic approaches targeting lipid metabolism, the CRC-derived cell models certainly have also some limitations. In future studies, it will be important to establish both biological significance and the mechanisms responsible for the observed up-regulation or down-regulation of individual PLs in colon tumor cells.

## Supporting information

S1 Fig Variability of primary values. Baseline description of primary data profiles (primary peak values, average from 3 replicates and corresponded standard error—SE). The variability of the primary estimates is associated (is growing) only with the value of peak area (A) but still with a small coefficient of variance. The analysis proved that standard error of the repeated estimates is fully random, not specifically associated with measured compounds or their characteristics, such as e.g. molecular weight (B).
(PDF)

S2 Fig Relation between molecular weights or number of double bonds peak areas in individual phospholipid classes. Cumulative peak areas (primary data) according to lipid species molecular weights and number of double (D) bonds within phosphatidylcholines (PC), phosphatidyletanolamines (PE), phosphatidylinositols (PI), and phosphatidylserines (PS) are shown for all colon epithelial cell lines. Molecular weight is the main covariate of the total PL content in all the cell lines; the biggest differences in PC, PE and PS amount among the cell lines are generated by the content of species with molecular weight in the range 680–830. Within this range, the differences among the lines in the total PL content are established and species with higher molecular weight than 830 do not further contribute to the differencing. On the contrary, the most discriminating PI species are those with MW>830. Increment in number of double bonds did not significantly influence changes in the total amount of PC, PE and PS classes—their content in all compared cell lines significantly increases due to contribution of PL compounds with low degree of saturation, i.e. with ≤ 1 double bond. Only PI species with higher double bond number (2–5) are also contributing significantly to total lipid mass.
(PDF)

S3 Fig Relation between molecular weights or number of double bonds and peak area in TAGs and CholE. Cumulative peak areas (primary data) according to lipid species molecular weights and number of double (D) bonds within triacylglycerols (TAG) and cholesterol-esters (CholE) are shown for all colon epithelial cell lines.
(PDF)

S4 Fig Comparison of PL profiles between patient-derived primary cells and NCM460/ SW480 cell lines. Relative distribution (i.e. sum of all shown MW species gives 100%) of specific PL species in non-tumor and tumor primary epithelial cells (mean value, n = 8) as well as non-tumor (NCM460) and tumor (SW480) derived cell lines. Carbon and double bond (DB) numbers are shown in parentheses. Only PL species, which were above detection limit both in patient’s samples and in cell lines are shown here.
(PDF)

S1 Table Analysis of variance of peak areas (log-scale). A detailed analysis of repeated estimates (3 independent repeats) of phospholipid profiles confirmed a high degree of repeatability of the experimental outcomes. Analysis of variance (performed on log-scaled peak areas) revealed coefficient of variance in the range of 16.1–22.1% which confirms effective normalization of the peak data base on logarithmic transformation. Random error exhausted only 0.09% of the overall experimental variance (calculated as a proportion of the total sum of squares). Furthermore, test of homogeneity of variance among compared cell lines proved acceptable homogeneity (*p* = 0.184) which enables a direct comparison of lipid profiles among lines.
(PDF)

S2 Table Suggested fatty acid (FA) pattern of phosphatidylcholine (PC), phosphatidylethanolamine (PE), phosphatidylserine (PS) and phosphatidylinositol (PI) species according to their molecular weights identified in colon cellular models. (PDF)

## List of abbreviations

APPI: atmospheric pressure photoionization
C: carbon
CholE: cholesteryl-ester
CRC: colorectal cancer
DB: double bond
EpCAM: epithelial cell adhesion molecule
ESI: electrospray ionization
FA: fatty acid
FBS: fetal bovine serum
HPLC: high performance liquid chromatography
LC-MS/MS: liquid chromatography-tandem mass spectrometry
MW: molecular weight
N: non-tumor cells
PC: phosphatidylcholine (diacylglycerophosphocholine)
PCA: principal component analysis
PE: phosphatidylethanolamine (diacylglycerophosphoethanolamine)
PI: phosphatidylinositol (diacylglycerophosphoinositol)
PL: phospholipid; PS, phosphatidylserine (diacylglycerophosphoserine)
PUFA: polyunsaturated fatty acid
RT: room temperature
T: tumor cells
T/N: tumor/non-tumor cell ratio
TAG: triacylglycerol

## References

[1] Grady WM, Carethers JM. Genomic and epigenetic instability in colorectal cancer pathogenesis. Gastroenterology. 2008;135(4):1079–99. 10.1053/j.gastro.2008.07.076 . 18773902 PMC2866182

[2] Yan G, Li L, Zhu B, Li Y. Lipidome in colorectal cancer. Oncotarget. 2016;7(22):33429–39. 10.18632/oncotarget.7960 . 26967051 PMC5078107

[3] Santos CR, Schulze A. Lipid metabolism in cancer. FEBS J. 2012;279(15):2610–23. 10.1111/j.1742-4658.2012.08644.x . 22621751

[4] Yaqoob P, Shaikh SR. The nutritional and clinical significance of lipid rafts. Curr Opin Clin Nutr Metab Care. 2010;13(2):156–66. 10.1097/MCO.0b013e328335725b . 20010096

[5] Bennett A, Civier A, Hensby CN, Melhuish PB, Stamford IF. Measurement of arachidonate and its metabolites extracted from human normal and malignant gastrointestinal tissues. Gut. 1987;28(3):315–8. 10.1136/gut.28.3.315 . 3106172 PMC1432696

[6] Fernández-Banares F, Esteve M, Navarro E, Cabre E, Boix J, Abad-Lacruz A, et al Changes of the mucosal n3 and n6 fatty acid status occur early in the colorectal adenoma-carcinoma sequence. Gut. 1996;38(2):254–9. 10.1136/gut.38.2.254 . 8801207 PMC1383033

[7] Neoptolemos JP, Husband D, Imray C, Rowley S, Lawson N. Arachidonic acid and docosahexaenoic acid are increased in human colorectal cancer. Gut. 1991;32(3):278–81. 10.1136/gut.32.3.278 . 1826490 PMC1378834

[8] Li F, Qin X, Chen H, Qiu L, Guo Y, Liu H, et al Lipid profiling for early diagnosis and progression of colorectal cancer using direct-infusion electrospray ionization Fourier transform ion cyclotron resonance mass spectrometry. Rapid Commun Mass Spectrom. 2013;27(1):24–34. 10.1002/rcm.6420 . 23239314

[9] Oraldi M, Trombetta A, Biasi F, Canuto RA, Maggiora M, Muzio G. Decreased polyunsaturated fatty acid content contributes to increased survival in human colon cancer. J Oncol. 2009;2009:867915 10.1155/2009/867915 . 19841681 PMC2762309

[10] Rakheja D, Kapur P, Hoang MP, Roy LC, Bennett MJ. Increased ratio of saturated to unsaturated C18 fatty acids in colonic adenocarcinoma: implications for cryotherapy and lipid raft function. Med Hypotheses. 2005;65(6):1120–3. 10.1016/j.mehy.2005.05.045 . 16084671

[11] Szachowicz-Petelska B, Sulkowski S, Figaszewski ZA. Altered membrane free unsaturated fatty acid composition in human colorectal cancer tissue. Mol Cell Biochem. 2007;294(1–2):237–42. 10.1007/s11010-006-9264-x . 16858511

[12] Zhang J, Zhang L, Ye X, Chen L, Gao Y, Kang JX, et al Characteristics of fatty acid distribution is associated with colorectal cancer prognosis. Prostaglandins Leukot Essent Fatty Acids. 2013;88(5):355–60. 10.1016/j.plefa.2013.02.005 . 23465412

[13] Baro L, Hermoso JC, Nunez MC, Jimenez-Rios JA, Gil A. Abnormalities in plasma and red blood cell fatty acid profiles of patients with colorectal cancer. Br J Cancer. 1998;77(11):1978–83. 10.1038/bjc.1998.328 . 9667678 PMC2150320

[14] Berstad P, Thiis-Evensen E, Vatn MH, Almendingen K. Fatty acids in habitual diet, plasma phospholipids, and tumour and normal colonic biopsies in young colorectal cancer patients. J Oncol. 2012;2012:254801 10.1155/2012/254801 . 23319946 PMC3540828

[15] Pot GK, Geelen A, van Heijningen EM, Siezen CL, van Kranen HJ, Kampman E. Opposing associations of serum n-3 and n-6 polyunsaturated fatty acids with colorectal adenoma risk: an endoscopy-based case-control study. Int J Cancer. 2008;123(8):1974–7. 10.1002/ijc.23729 . 18661525

[16] Bandu R, Mok HJ, Kim KP. Phospholipids as cancer biomarkers: Mass spectrometry-based analysis. Mass Spectrom Rev. 2018;37(2):107–38. 10.1002/mas.21510 . 27276657

[17] Azordegan N, Fraser V, Le K, Hillyer LM, Ma DW, Fischer G, et al Carcinogenesis alters fatty acid profile in breast tissue. Mol Cell Biochem. 2013;374(1–2):223–32. 10.1007/s11010-012-1523-4 . 23180247

[18] Bestard-Escalas J, Garate J, Maimo-Barcelo A, Fernandez R, Lopez DH, Lage S, et al Lipid fingerprint image accurately conveys human colon cell pathophysiologic state: A solid candidate as biomarker. Biochim Biophys Acta. 2016;1861(12 Pt A):1942–50. 10.1016/j.bbalip.2016.09.013 . 27663183

[19] Fernandis AZ, Wenk MR. Lipid-based biomarkers for cancer. J Chromatogr B Analyt Technol Biomed Life Sci. 2009;877(26):2830–5. 10.1016/j.jchromb.2009.06.015 . 19570730

[20] Perrotti F, Rosa C, Cicalini I, Sacchetta P, Del Boccio P, Genovesi D, et al Advances in Lipidomics for Cancer Biomarkers Discovery. Int J Mol Sci. 2016;17(12). 10.3390/ijms17121992 . 27916803 PMC5187792

[21] Hofmanová J, Slavík J, Ovesná P, Tylichová Z, Vondráček J, Straková N, et al Dietary fatty acids specifically modulate phospholipid pattern in colon cells with distinct differentiation capacities. Eur J Nutr. 2017;56(4):1493–508. 10.1007/s00394-016-1196-y . 26983609

[22] Leyssens C, Marien E, Verlinden L, Derua R, Waelkens E, Swinnen JV, et al Remodeling of phospholipid composition in colon cancer cells by 1alpha,25(OH)2D3 and its analogs. J Steroid Biochem Mol Biol. 2015;148:172–8. 10.1016/j.jsbmb.2015.01.018 . 25625664

[23] Marien E, Meister M, Muley T, Fieuws S, Bordel S, Derua R, et al Non-small cell lung cancer is characterized by dramatic changes in phospholipid profiles. Int J Cancer. 2015;137(7):1539–48. 10.1002/ijc.29517 . 25784292 PMC4503522

[24] Williams AC, Harper SJ, Paraskeva C. Neoplastic transformation of a human colonic epithelial cell line: in vitro evidence for the adenoma to carcinoma sequence. Cancer Res. 1990;50(15):4724–30. . 2369746

[25] Paraskeva C, Finerty S, Mountford RA, Powell SC. Specific cytogenetic abnormalities in two new human colorectal adenoma-derived epithelial cell lines. Cancer Res. 1989;49(5):1282–6. . 2917357

[26] Souček K, Gajdůšková P, Brázdová M, Hýžd'alová M, Kočí L, Vydra D, et al Fetal colon cell line FHC exhibits tumorigenic phenotype, complex karyotype, and TP53 gene mutation. Cancer genetics and cytogenetics. 2010;197(2):107–16. 10.1016/j.cancergencyto.2009.11.009 . 20193843

[27] Hague A, Hanlon K, Paraskeva C. Clonal evolution and tumor progression in 2 human colorectal adenoma-derived cell-lines invitro—the involvement of chromosome-1 abnormalities. Int J Oncol. 1992;1(2):201–8. 10.3892/ijo.1.2.201 . 21584532

[28] Knutsen T, Padilla-Nash HM, Wangsa D, Barenboim-Stapleton L, Camps J, McNeil N, et al Definitive molecular cytogenetic characterization of 15 colorectal cancer cell lines. Genes Chromosomes Cancer. 2010;49(3):204–23. 10.1002/gcc.20730 . 19927377 PMC2818350

[29] Chen P, Luo X, Nie P, Wu B, Xu W, Shi X, et al CQ synergistically sensitizes human colorectal cancer cells to SN-38/CPT-11 through lysosomal and mitochondrial apoptotic pathway via p53-ROS cross-talk. Free Rad Biol Med. 2017;104:280–97. 10.1016/j.freeradbiomed.2017.01.033 . 28131902

[30] Williams AC, Browne SJ, Manning AM, Daffada P, Collard TJ, Paraskeva C. Transfection and expression of mutant p53 protein does not alter the in vivo or in vitro growth characteristics of the AA/C1 human adenoma derived cell line, including sensitivity to transforming growth factor-beta 1. Oncogene. 1994;9(5):1479–85. . 8152811

[31] Ahmed D, Eide PW, Eilertsen IA, Danielsen SA, Eknaes M, Hektoen M, et al Epigenetic and genetic features of 24 colon cancer cell lines. Oncogenesis. 2013;2:e71 10.1038/oncsis.2013.35 . 24042735 PMC3816225

[32] Browne SJ, Williams AC, Hague A, Butt AJ, Paraskeva C. Loss of APC protein expressed by human colonic epithelial cells and the appearance of a specific low-molecular-weight form is associated with apoptosis in vitro. Int J Cancer. 1994;59(1):56–64. 10.1002/ijc.2910590113 . 7927905

[33] Rowan AJ, Lamlum H, Ilyas M, Wheeler J, Straub J, Papadopoulou A, et al APC mutations in sporadic colorectal tumors: A mutational "hotspot" and interdependence of the "two hits". Proc Natl Acad Sci U S A. 2000;97(7):3352–7. 10.1073/pnas.97.7.3352 . 10737795 PMC16243

[34] Ikediobi ON, Davies H, Bignell G, Edkins S, Stevens C, O'Meara S, et al Mutation analysis of 24 known cancer genes in the NCI-60 cell line set. Mol Cancer Ther. 2006;5(11):2606–12. 10.1158/1535-7163.MCT-06-0433 . 17088437 PMC2705832

[35] Greenhough A, Wallam CA, Hicks DJ, Moorghen M, Williams AC, Paraskeva C. The proapoptotic BH3-only protein Bim is downregulated in a subset of colorectal cancers and is repressed by antiapoptotic COX-2/PGE(2) signalling in colorectal adenoma cells. Oncogene. 2010;29(23):3398–410. 10.1038/onc.2010.94 . 20348947 PMC2883743

[36] Minoo P, Moyer MP, Jass JR. Role of BRAF-V600E in the serrated pathway of colorectal tumourigenesis. J Pathol. 2007;212(2):124–33. 10.1002/path.2160 . 17427169

[37] Céspedes MV, Espina C, García-Cabezas MA, Trias M, Boluda A, Gómez del Pulgar MT, et al Orthotopic microinjection of human colon cancer cells in nude mice induces tumor foci in all clinically relevant metastatic sites. Am J Pathol. 2007;170(3):1077–85. 10.2353/ajpath.2007.060773 . 17322390 PMC1864873

[38] Doria ML, Ribeiro AS, Wang J, Cotrim CZ, Domingues P, Williams C, et al Fatty acid and phospholipid biosynthetic pathways are regulated throughout mammary epithelial cell differentiation and correlate to breast cancer survival. FASEB J. 2014;28(10):4247–64. 10.1096/fj.14-249672 . 24970396

[39] Pakiet A, Kobiela J, Stepnowski P, Sledzinski T, Mika A. Changes in lipids composition and metabolism in colorectal cancer: a review. Lipids Health Dis. 2019;18(1):29 10.1186/s12944-019-0977-8 . 30684960 PMC6347819

[40] Dobrzynska I, Szachowicz-Petelska B, Sulkowski S, Figaszewski Z. Changes in electric charge and phospholipids composition in human colorectal cancer cells. Mol Cell Biochem. 2005;276(1–2):113–9. 10.1007/s11010-005-3557-3 . 16132692

[41] Dueck DA, Chan M, Tran K, Wong JT, Jay FT, Littman C, et al The modulation of choline phosphoglyceride metabolism in human colon cancer. Mol Cell Biochem. 1996;162(2):97–103. 10.1007/bf00227535 . 8905631

[42] Kurabe N, Hayasaka T, Ogawa M, Masaki N, Ide Y, Waki M, et al Accumulated phosphatidylcholine (16:0/16:1) in human colorectal cancer; possible involvement of LPCAT4. Cancer Sci. 2013;104(10):1295–302. 10.1111/cas.12221 . 23815430 PMC7656554

[43] Li SY, Yu B, An P, Liang ZJ, Yuan SJ, Cai HY. Effects of cell membrane phospholipid level and protein kinase C isoenzyme expression on hepatic metastasis of colorectal carcinoma. Hepatobiliary Pancreat Dis Int. 2004;3(3):411–6. . 15313680

[44] Doria ML, Cotrim CZ, Simoes C, Macedo B, Domingues P, Domingues MR, et al Lipidomic analysis of phospholipids from human mammary epithelial and breast cancer cell lines. J Cell Physiol. 2013;228(2):457–68. 10.1002/jcp.24152 . 22767159

[45] Baeuerle PA, Gires O. EpCAM (CD326) finding its role in cancer. Br J Cancer. 2007;96(3):417–23. 10.1038/sj.bjc.6603494 . 17211480 PMC2360029

[46] Fhaner CJ, Liu S, Ji H, Simpson RJ, Reid GE. Comprehensive lipidome profiling of isogenic primary and metastatic colon adenocarcinoma cell lines. Anal Chem. 2012;84(21):8917–26. 10.1021/ac302154g . 23039336 PMC3491142

[47] Luo X, Cheng C, Tan Z, Li N, Tang M, Yang L, et al Emerging roles of lipid metabolism in cancer metastasis. Mol Cancer. 2017;16(1):76 10.1186/s12943-017-0646-3 . 28399876 PMC5387196

[48] Doria ML, Cotrim Z, Macedo B, Simoes C, Domingues P, Helguero L, et al Lipidomic approach to identify patterns in phospholipid profiles and define class differences in mammary epithelial and breast cancer cells. Breast Cancer Res Treat. 2012;133(2):635–48. 10.1007/s10549-011-1823-5 . 22037781

[49] Johnson CH, Santidrian AF, LeBoeuf SE, Kurczy ME, Rattray NJW, Rattray Z, et al Metabolomics guided pathway analysis reveals link between cancer metastasis, cholesterol sulfate, and phospholipids. Cancer Metab. 2017;5:9 10.1186/s40170-017-0171-2 . 29093815 PMC5663111

[50] Kim HY, Lee H, Kim SH, Jin H, Bae J, Choi HK. Discovery of potential biomarkers in human melanoma cells with different metastatic potential by metabolic and lipidomic profiling. Sci Rep. 2017;7(1):8864 10.1038/s41598-017-08433-9 . 28821754 PMC5562697

[51] Kim HY, Lee KM, Kim SH, Kwon YJ, Chun YJ, Choi HK. Comparative metabolic and lipidomic profiling of human breast cancer cells with different metastatic potentials. Oncotarget. 2016;7(41):67111–28. 10.18632/oncotarget.11560 . 27564096 PMC5341861

[52] Peng W, Tan S, Xu Y, Wang L, Qiu D, Cheng C, et al LCMS/MS metabolome analysis detects the changes in the lipid metabolic profiles of dMMR and pMMR cells. Oncol Rep. 2018;40(2):1026–34. 10.3892/or.2018.6510 . 29989648

[53] Sampaio JL, Gerl MJ, Klose C, Ejsing CS, Beug H, Simons K, et al Membrane lipidome of an epithelial cell line. Proc Natl Acad Sci U S A. 2011;108(5):1903–7. 10.1073/pnas.1019267108 . 21245337 PMC3033259

[54] Tylichová Z, Straková N, Vondráček J, Vaculová AH, Kozubík A, Hofmanová J. Activation of autophagy and PPARgamma protect colon cancer cells against apoptosis induced by interactive effects of butyrate and DHA in a cell type-dependent manner: The role of cell differentiation. J Nutr Biochem. 2017;39:145–55. 10.1016/j.jnutbio.2016.09.006 . 27840291

[55] Ridgway ND. The role of phosphatidylcholine and choline metabolites to cell proliferation and survival. Crit Rev Biochem Mol Biol. 2013;48(1):20–38. 10.3109/10409238.2012.735643 . 23350810

[56] Mirnezami R, Spagou K, Vorkas PA, Lewis MR, Kinross J, Want E, et al Chemical mapping of the colorectal cancer microenvironment via MALDI imaging mass spectrometry (MALDI-MSI) reveals novel cancer-associated field effects. Mol Oncol. 2014;8(1):39–49. 10.1016/j.molonc.2013.08.010 . 24112879 PMC5528498

[57] Guo S, Wang Y, Zhou D, Li Z. Significantly increased monounsaturated lipids relative to polyunsaturated lipids in six types of cancer microenvironment are observed by mass spectrometry imaging. Sci Rep. 2014;4:5959 10.1038/srep05959 . 25091112 PMC4121604

[58] Thomas A, Patterson NH, Marcinkiewicz MM, Lazaris A, Metrakos P, Chaurand P. Histology-driven data mining of lipid signatures from multiple imaging mass spectrometry analyses: application to human colorectal cancer liver metastasis biopsies. Anal Chem. 2013;85(5):2860–6. 10.1021/ac3034294 . 23347294

[59] Goto T, Terada N, Inoue T, Kobayashi T, Nakayama K, Okada Y, et al Decreased expression of lysophosphatidylcholine (16:0/OH) in high resolution imaging mass spectrometry independently predicts biochemical recurrence after surgical treatment for prostate cancer. Prostate. 2015;75(16):1821–30. 10.1002/pros.23088 . 26332786

[60] Kawashima M, Iwamoto N, Kawaguchi-Sakita N, Sugimoto M, Ueno T, Mikami Y, et al High-resolution imaging mass spectrometry reveals detailed spatial distribution of phosphatidylinositols in human breast cancer. Cancer Sci. 2013;104(10):1372–9. 10.1111/cas.12229 . 23837649 PMC7656533

[61] Koeberle A, Shindou H, Koeberle SC, Laufer SA, Shimizu T, Werz O. Arachidonoyl-phosphatidylcholine oscillates during the cell cycle and counteracts proliferation by suppressing Akt membrane binding. Proc Natl Acad Sci U S A. 2013;110(7):2546–51. 10.1073/pnas.1216182110 . 23359699 PMC3574958

[62] Vance JE, Tasseva G. Formation and function of phosphatidylserine and phosphatidylethanolamine in mammalian cells. Biochim Biophys Acta. 2013;1831(3):543–54. 10.1016/j.bbalip.2012.08.016 . 22960354

