# Supplementary material for: Phospholipid profiling enables to discriminate tumor- and non-tumor-derived human colon epithelial cells: Phospholipidome similarities and differences in colon cancer cell lines and in patient-derived cell samples
Source: PLoS One. 2020 Jan 30;15(1):e0228010. doi: 10.1371/journal.pone.0228010 (PMC6992008; doi:10.1371/journal.pone.0228010)
Supplement: S1 Fig — Baseline description of primary data profiles (primary peak values, average from 3 replicates and corresponded standard error—SE). The variability of the primary estimates is associated (is growing) only with the value of peak area (A) but still with a small coefficient of variance. The analysis proved that standard error of the repeated estimates is fully random, not specifically associated with measured compounds or their characteristics, such as e.g. molecular weight (B). (PDF) [file pone.0228010.s001.pdf]

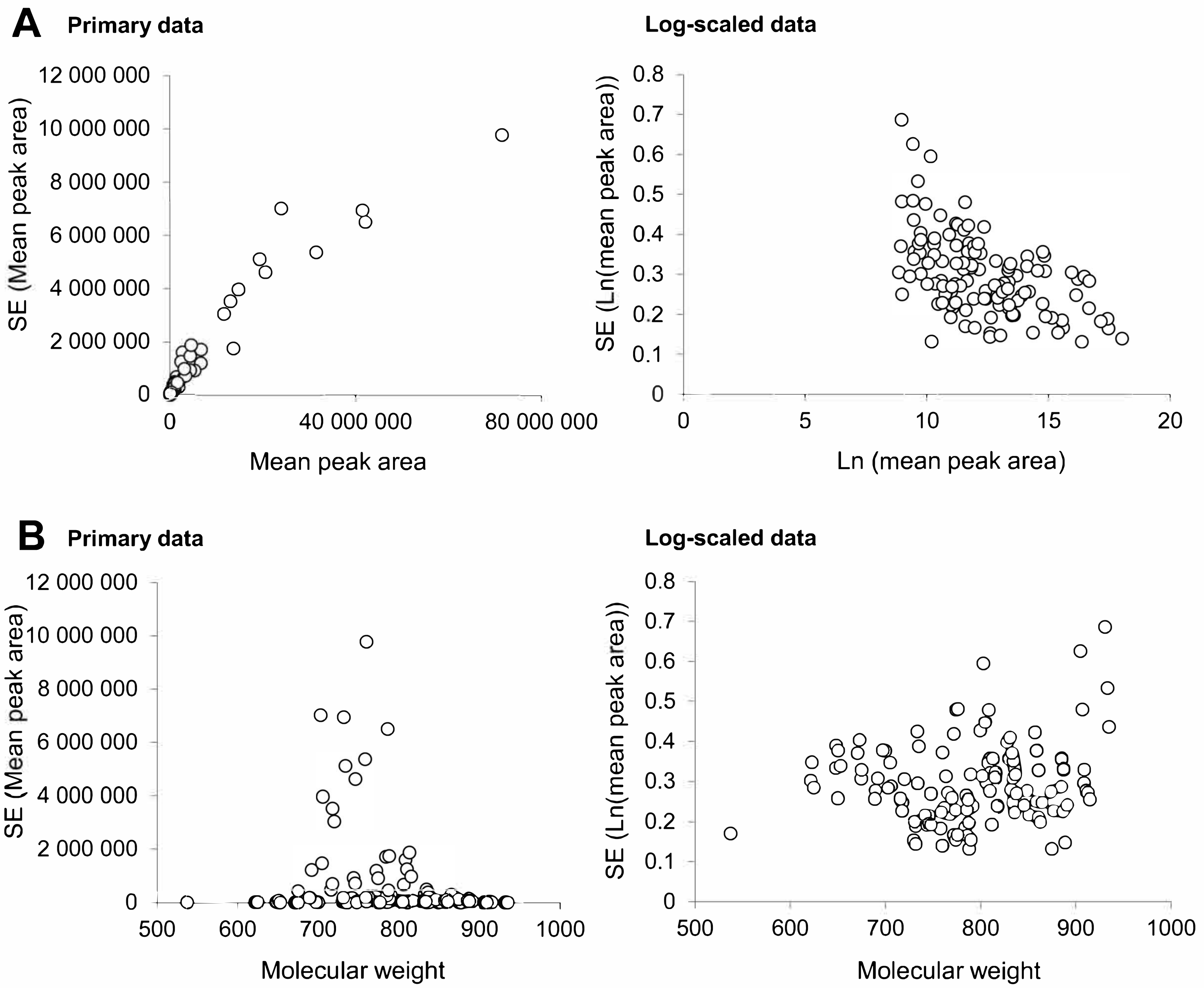

S1 Fig. Variability of primary values

Baseline description of primary data profiles (primary peak values, average from 3 replicates and corresponded standard error – SE). The variability of the primary estimates is associated (is growing) only with the value of peak area (A) but still with a small coefficient of variance. The analysis proved that standard error of the repeated estimates is fully random, not specifically associated with measured compounds or their characteristics, such as e.g. molecular weight (B).
