# Supplementary material for: Phospholipid profiling enables to discriminate tumor- and non-tumor-derived human colon epithelial cells: Phospholipidome similarities and differences in colon cancer cell lines and in patient-derived cell samples
Source: PLoS One. 2020 Jan 30;15(1):e0228010. doi: 10.1371/journal.pone.0228010 (PMC6992008; doi:10.1371/journal.pone.0228010)
Supplement: S2 Fig — Cumulative peak areas (primary data) according to lipid species molecular weights and number of double (D) bonds within phosphatidylcholines (PC), phosphatidyletanolamines (PE), phosphatidylinositols (PI), and phosphatidylserines (PS) are shown for all colon epithelial cell lines. Molecular weight is the main covariate of the total PL content in all the cell lines; the biggest differences in PC, PE and PS amount among the cell lines are generated by the content of species with molecular weight in the range 680–830. Within this range, the differences among the lines in the total PL content are established and species with higher molecular weight than 830 do not further contribute to the differencing. On the contrary, the most discriminating PI species are those with MW>830. Increment in number of double bonds did not significantly influence changes in the total amount of PC, PE and PS classes—their content in all compared cell lines significantly increases due to contribution of PL compounds with low degree of saturation, i.e. with ≤ 1 double bond. Only PI species with higher double bond number (2–5) are also contributing significantly to total lipid mass. (PDF) [file pone.0228010.s002.pdf]

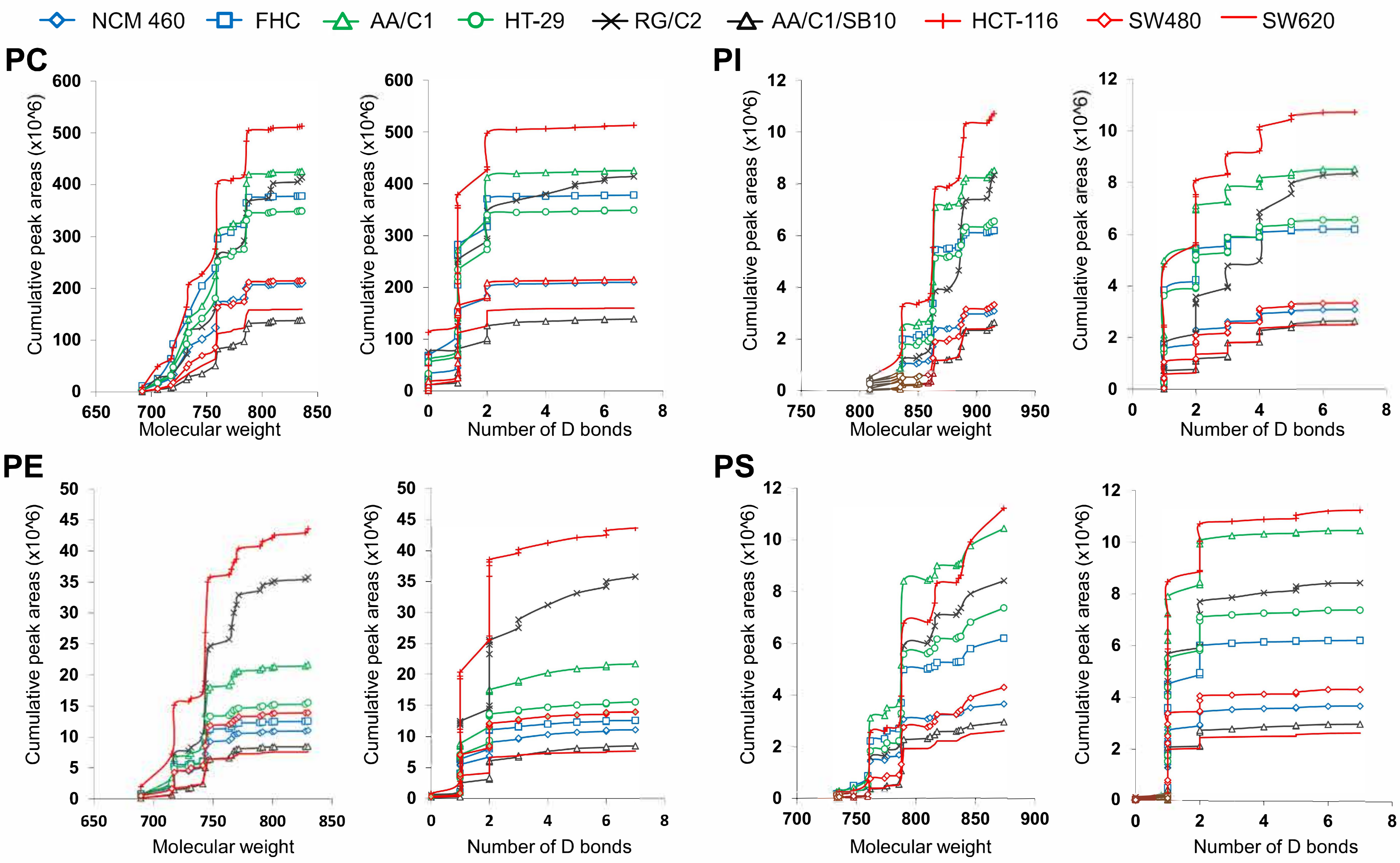

S2 Fig. Relation between molecular weights or number of double (D) bonds peak areas in individual phospholipid classes.
Cumulative peak areas (primary data) according to lipid species molecular weights and number of double (D) bonds within phosphatidylcholines (PC), phosphatidyletanolamines (PE), phosphatidylinositols (PI), and phosphatidylserines (PS) are shown for all colon epithelial cell lines.

Molecular weight is the main covariate of the total PL content in all the cell lines; the biggest differences in PC, PE and PS amount among the cell lines are generated by the content of species with molecular weight in the range 680 – 830. Within this range, the differences among the lines in the total PL content are established and species with higher molecular weight than 830 do not further contribute to the differencing. On the contrary, the most discriminating PI species are those with MW>830.

Increment in number of double bonds did not significantly influence changes in the total amount of PC, PE and PS classes – their content in all compared cell lines significantly increases due to contribution of PL compounds with low degree of saturation, i.e. with ≤ 1 DB. Only PI species with higher double bonds (2–5) are also contributing significantly to total lipid mass.
