# Supplementary material for: Phospholipid profiling enables to discriminate tumor- and non-tumor-derived human colon epithelial cells: Phospholipidome similarities and differences in colon cancer cell lines and in patient-derived cell samples
Source: PLoS One. 2020 Jan 30;15(1):e0228010. doi: 10.1371/journal.pone.0228010 (PMC6992008; doi:10.1371/journal.pone.0228010)
Supplement: S3 Fig — Cumulative peak areas (primary data) according to lipid species molecular weights and number of double (D) bonds within triacylglycerols (TAG) and cholesterol-esters (CholE) are shown for all colon epithelial cell lines. (PDF) [file pone.0228010.s003.pdf]

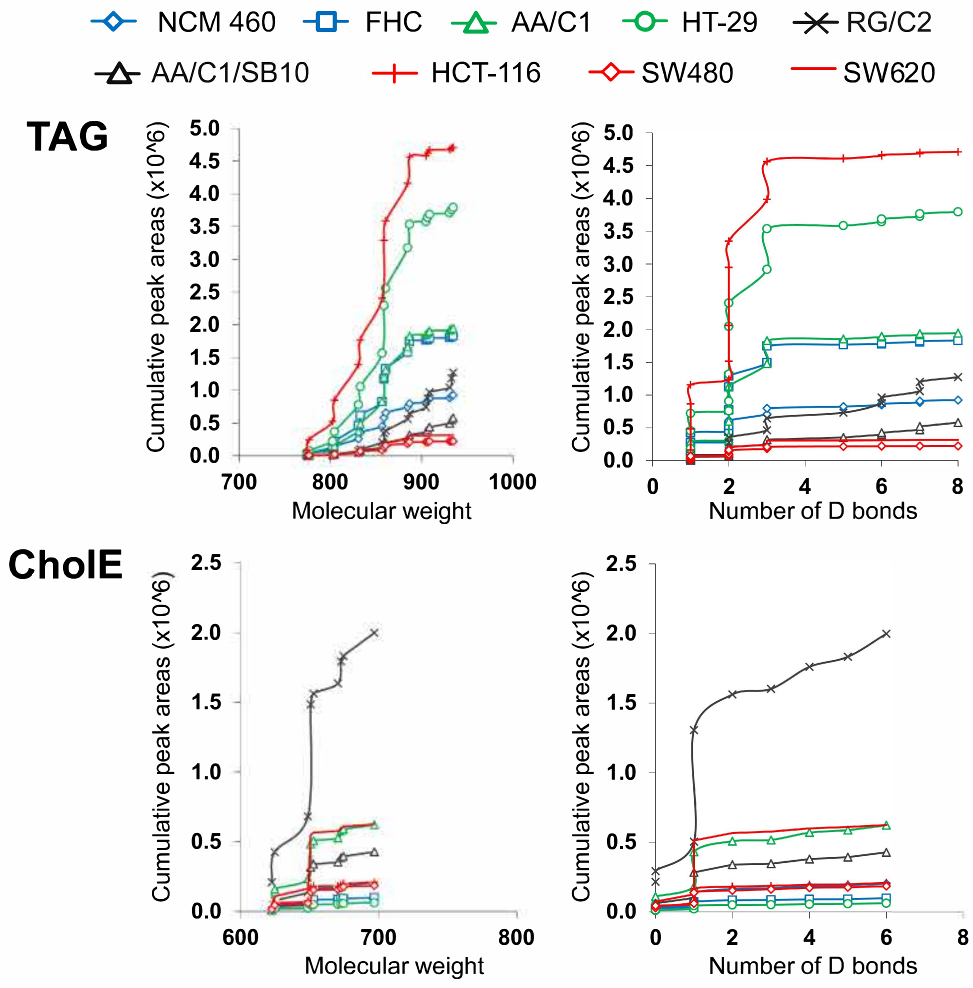

S3 Fig. Relation between molecular weights or number of double (D) bonds and peak area in TAGs and CholE. Cumulative peak areas (primary data) according to lipid species molecular weights and number of double (D) bonds within triacylglycerols (TAG) and cholesterol-esters (CholE) are shown for all colon epithelial cell lines.
