# Supplementary material for: Phospholipid profiling enables to discriminate tumor- and non-tumor-derived human colon epithelial cells: Phospholipidome similarities and differences in colon cancer cell lines and in patient-derived cell samples
Source: PLoS One. 2020 Jan 30;15(1):e0228010. doi: 10.1371/journal.pone.0228010 (PMC6992008; doi:10.1371/journal.pone.0228010)
Supplement: S4 Fig — Relative distribution (i.e. sum of all shown MW species gives 100%) of specific PL species in non-tumor and tumor primary epithelial cells (mean value, n = 8) as well as non-tumor (NCM460) and tumor (SW480) derived cell lines. Carbon and double bond (DB) numbers are shown in parentheses. Only PL species, which were above detection limit both in patient’s samples and in cell lines are shown here. (PDF) [file pone.0228010.s004.pdf]

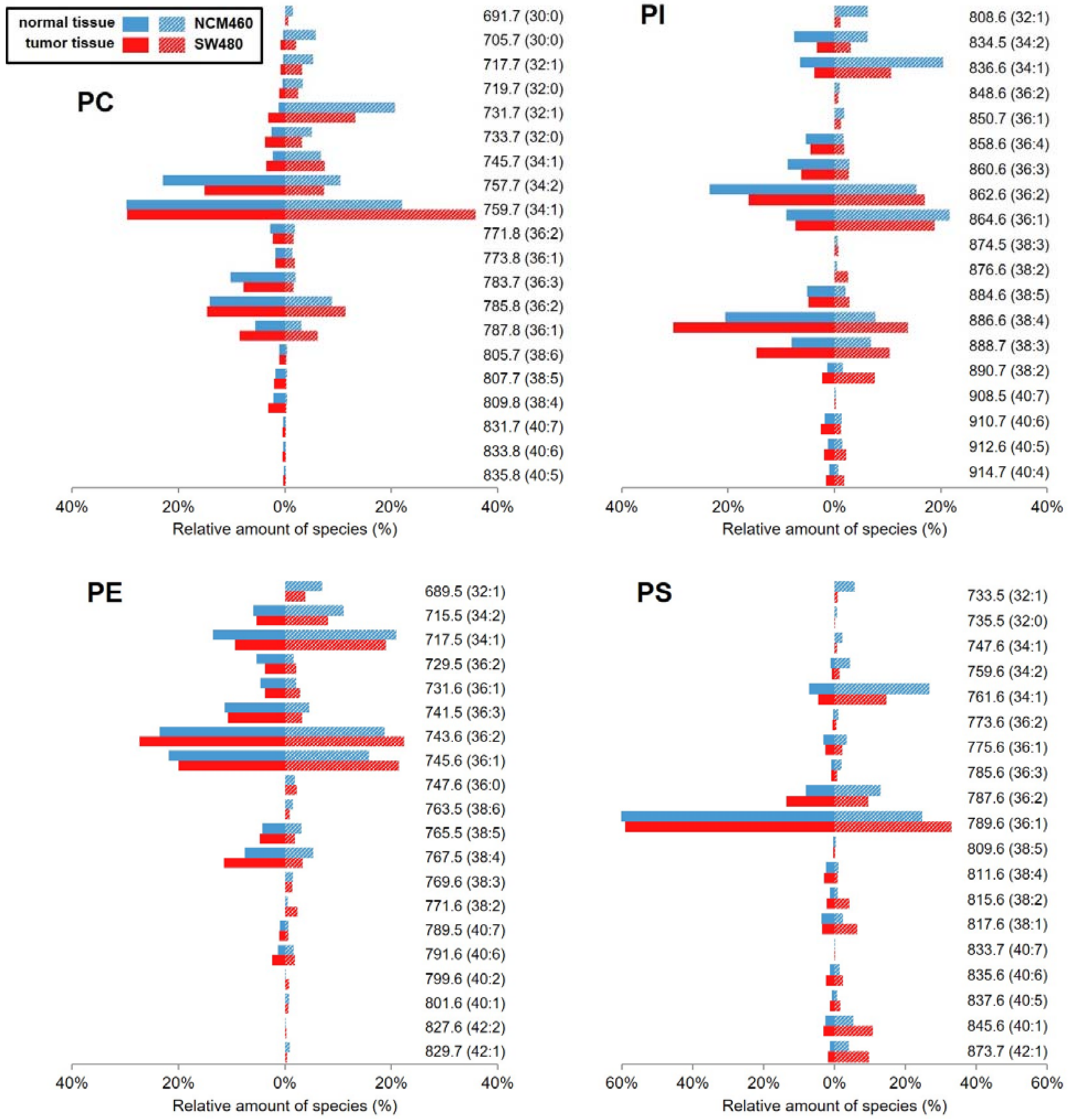

Supplement Figure 4 Comparison of PL profiles between patient-derived primary cells and NCM460/SW480 cell lines. Relative distribution (i.e. sum of all shown MW species gives 100%) of specific PL species in non-tumor and tumor primary epithelial cells (mean value, n = 8) as well as non-tumor (NCM460) and tumor (SW480) derived cell lines. Carbon and double bond (DB) numbers are shown in parentheses. Only PL species, which were above detection limit both in patient's samples and in cell lines are shown here.
