# Supplementary material for: Phospholipid profiling enables to discriminate tumor- and non-tumor-derived human colon epithelial cells: Phospholipidome similarities and differences in colon cancer cell lines and in patient-derived cell samples
Source: PLoS One. 2020 Jan 30;15(1):e0228010. doi: 10.1371/journal.pone.0228010 (PMC6992008; doi:10.1371/journal.pone.0228010)
Supplement: S1 Table — A detailed analysis of repeated estimates (3 independent repeats) of phospholipid profiles confirmed a high degree of repeatability of the experimental outcomes. Analysis of variance (performed on log-scaled peak areas) revealed coefficient of variance in the range of 16.1–22.1% which confirms effective normalization of the peak data base on logarithmic transformation. Random error exhausted only 0.09% of the overall experimental variance (calculated as a proportion of the total sum of squares). Furthermore, test of homogeneity of variance among compared cell lines proved acceptable homogeneity (p = 0.184) which enables a direct comparison of lipid profiles among lines. (PDF) [file pone.0228010.s005.pdf]

Suppl. Table 1: Analysis of variance of peak areas (log-scale)

Source	d.f.	SS	SS (%)	MS	p	Coef. variance[1]	Homogeneity of variance[2]
Line	8	1094.88	6.05%	136.86	<0.001	16.3% - 20.9%	F=1.42 *p*=0.184
Compound	120	15484.75	85.60%	129.04	<0.001	2.2% - 22.1%	
Line x Compound	960	1490.69	8.24%	1.55	<0.001		
Compound x Replicate	242	2.35	0.01%	0.01	0.106		
Error	1935	16.76	0.09%	0.01			
Total	3266	18089.44					

A detailed analysis of repeated estimates (3 independent repeats) of phospholipid profiles confirmed a high degree of repeatability of the experimental outcomes. Analysis of variance (performed on log-scaled peak areas) revealed coefficient of variance in the range of 16.1 – 22.1% which confirms effective normalization of the peak data base on logarithmic transformation. Random error exhausted only 0.09% of the overall experimental variance (calculated as a proportion of the total sum of squares). Furthermore, test of homogeneity of variance among compared cell lines proved acceptable homogeneity (p = 0.184) which enables a direct comparison of lipid profiles among lines.

[1] range of coefficients of variance in given stratification

[2] Levene test

d.f. degrees of freedom; SS sum of squares; MS mean sum of squares
