# Supplementary material for: Phospholipid profiling enables to discriminate tumor- and non-tumor-derived human colon epithelial cells: Phospholipidome similarities and differences in colon cancer cell lines and in patient-derived cell samples
Source: PLoS One. 2020 Jan 30;15(1):e0228010. doi: 10.1371/journal.pone.0228010 (PMC6992008; doi:10.1371/journal.pone.0228010)
Supplement: S2 Table — (PDF) [file pone.0228010.s006.pdf]

Suppl. Table 2: Suggested fatty acid (FA) pattern of phosphatidylcholine (PC), phosphatidylethanolamine (PE), phosphatidylserine (PS) and phosphatidylinositol (PI) species according to their molecular weights identified in colon cellular models.

**MW**	***m/z*** **[M+H]+**	**Total FA carbons and double bonds**	**Fatty acid variations of respective lipid species**			
**PC**						
691.7	692.7	30:0	14:0(pla)//16:0			
705.7	706.7	30:0	12:0//18:0	14:0//16:0		
717.7	718.7	32:1	14:0(pla)//18:1			
719.7	720.7	32:0	14:0(pla)//18:0			
731.7	732.7	32:1	14:0//18:1	16:0//16:1		
733.7	734.7	32:0	14:0//18:0	16:0//16:0		
745.7	746.7	34:1	14:0(pla)//20:1	16:0(pla)//18:1		
757.7	758.7	34:2	16:0//18:2	16:1//18:1		
759.7	760.7	34:1	16:0//18:1	16:1//18:0		
771.8	772.8	36:2	14:0(pla)//22:2	16:0(pla)//20:2	18:0(pla)//18:2	
773.8	774.8	36:1	14:0(pla)//22:1	16:0(pla)//20:1	18:0(pla)//18:1	
783.7	784.7	36:3	16:0//20:3	18:1//18:2	18:0//18:3	
785.8	786.8	36:2	16:1//20:1	18:0//18:2	18:1//18:1	
787.8	788.8	36:1	16:0//20:1	16:1//20:0	18:1//18:0	
805.7	806.7	38:6	18:2//20:4	18:3//20:3	18:1//20:5	
807.7	808.7	38:5	18:1//20:4	18:2//20:3	18:3//20:2	18:0//20:5
809.8	810.8	38:4	18:0//20:4	18:3//20:1	18:2//20:2	18:1//20:3
831.7	832.7	40:7	20:4//20:3	20:5//20:2		
833.8	834.8	40:6	20:3//20:3	20:4//20:2	20:5//20:1	
835.8	836.8	40:5	20:1//20:4	20:2//20:3	20:0//20:5	
**MW**	***m/z*** **[M+H]+**	**Total FA carbons and double bonds**	**Fatty acid variations of respective lipid species**			
**PE**						
689.5	690.5	32:1	16:0//16:1	14:0//18:1		
715.5	716.5	34:2	16:0//18:2	16:1//18:1		
717.5	718.5	34:1	16:0//18:1	16:1//18:0		
729.5	730.5	36:2	14:0ple//22:1	16:0ple//20:1	18:0ple//18:1	
731.6	732.6	36:1	14:0ple//22:0	16:0ple//20:0	18:0ple//18:0	
741.5	742.5	36:3	16:0//20:3	18:1//18:2	18:3//18:0	
743.6	744.6	36:2	20:1//16:1	18:0//18:2	18:1//18:1	
745.6	746.6	36:1	20:1//16:0	20:0//16:1	18:0//18:1	
747.6	748.6	36:0	20:0//16:0	18:0//18:0		
763.5	764.5	38:6	20:4//18:2	20:3//18:3		
765.5	766.5	38:5	20:4//18:1	20:3//18:2		
767.5	768.5	38:4	20:4//18:0	20:1//18:3	20:3//18:1	
769.6	770.6	38:3	20:1//18:2	20:0//18:3	20:3//18:0	
771.6	772.6	38:2	22:1//16:1	20:1//18:1	20:0//18:2	
789.5	790.5	40:7	20:4//20:3			
791.6	792.6	40:6	20:3//20:3			
799.6	800.6	40:2	22:0//18:2	20:1//20:1	22:1//18:1	24:1//16:1
801.6	802.6	40:1	20:0//20:1	22:1//16:0	24:1//16:0	22:0//18:1
827.6	828.6	42:2	22:1//20:1	24:1//18:1	24:0//18:2	
829.7	830.7	42:1	22:1//20:0	22:0//20:1	24:0//18:1	24:1//18:0

MW	*m/z* [M+H]+	Total FA carbons and double bonds	Fatty acid variations of respective lipid species			
**PI**						
808.6	826.6	32:1	16:0//16:1	14:0//18:1		
834.5	852.5	34:2	16:0//18:2	16:1//18:0		
836.6	854.6	34:1	16:0//18:1	16:1//18:0		
848.6	866.6	36:2	18:0pla//18:2	16:0pla//20:2	14:0pla//22:2	
850.7	868.7	36:1	16:0pla//20:1	14:0pla//22:1	18:0pla//18:1	
858.6	876.6	36:5	18:2//18:2	16:0//20:4	16:1//20:3	
860.6	878.6	36:3	18:1//18:2	16:0//20:3	18:0//18:3	
862.6	880.6	36:2	20:1//16:1	18:0//18:2	18:1//18:1	
864.6	882.6	36:1	20:1//16:0	16:1//20:0	18:0//18:1	
874.5	892.5	38:3	20:0pla//18:3	18:0pla//20:3	16:0pla//22:3	
876.6	894.6	38:2	20:0pla//18:2	18:0pla//22:2		
884.6	902.6	38:5	18:1//20:4	18:2//20:3	18:0//20:5	16:0//22:5
886.6	904.6	38:4	18:0//20:4	18:1//20:3	18:2//20:2	16:0//22:4
888.7	906.7	38:3	18:0//20:3	18:1//20:2	18:2//20:1	16:0//22:3
890.7	908.7	38:2	18:0//20:2	18:1//20:1	18:2//20:0	16:0//22:2
908.5	926.5	40:7	20:4//20:3	18:1//22:6		
910.7	928.7	40:6	20:3//20:3	20:2//20:4	18:0//22:6	
912.6	930.6	40:5	20:0//20:5	20:1//20:4	20:2//20:3	
914.7	932.7	40:4	20:0//20:4	20:1//20:3	20:2//20:2	18:0//22:4

MW	*m/z* [M+NH4]+	Total FA carbons and double bonds	Fatty acid variations of respective lipid species			
**PS**						
733.5	734.5	32:1	16:0//16:1	14:0//18:1		
735.5	736.5	32:0	16:0//16:0	14:0//18:0		
747.6	748.6	34:1	16:0pla//18:1	18:0pla//16:1		
759.6	760.6	34:2	16:0//18:2	16:1//18:1		
761.6	762.6	34:1	18:0//18:1	16:1//18:0		
773.6	774.6	36:2	18:0pla//18:2	16:0pla//20:2		
775.6	776.6	36:1	16:0pla//20:1	20:0pla//16:1	18:0pla//18:1	
785.6	786.6	36:3	20:2//16:1	18:1//18:2	18:0//18:3	20:3//16:0
787.6	788.6	36:2	20:1//16:1	18:0//18:2	18:1//18:1	20:2//16:0
789.6	790.6	36:1	20:1//16:0	20:0//16:1	18:0//18:1	
809.6	810.6	38:5	20:4//18:1	20:5//18:0	22:4//16:1	22:5//16:0
811.6	812.6	38:4	20:4//18:0	20:3//18:1	22:3//16:1	22:4//16:0
815.6	816.6	38:2	20:2//18:0	20:1//18:1	22:1//16:1	22:2//16:0
817.6	818.6	38:1	20:1//18:0	20:0//18:1	22:0//16:1	22:1//16:0
833.7	834.7	40:7	20:4//20:3	22:6//18:1		
835.6	836.6	40:6	20:3//20:3	22:6//18:0	22:5//18:1	
837.6	838.6	40:5	20:1//20:4	22:5//18:0	22:4//18:1	
845.6	846.6	40:1	20:0//20:1	22:1//18:0	24:1//16:0	22:0//18:1
873.7	874.7	42:1	22:1//20:0	22:0//20:1	24:0//18:1	24:1//18:0

Annotation:

fatty acid annotation: x:y // x:y - x=carbon number; y=double bond number;

fatty acids are attached via ester bonds in most of the analyzed species: -O-CO-R;

ple - fatty acid attached via vinyl bond: -O-CH=CH-R [1 of 2];

pla - fatty acid attached via alkyl ether bond: -O-R [1 of 2]
